# Perceptions of advice for acute low back pain: a content analysis of qualitative data collected in a randomised experiment

**DOI:** 10.1136/bmjopen-2023-079070

**Published:** 2024-07-23

**Authors:** Lidiya Augustine, Joshua Zadro, Christopher Maher, Adrian C Traeger, Caitlin Jones, Courtney A West, Jingjing Yang, Mary O'Keeffe, Hazel Jenkins, James H McAuley, Giovanni E Ferreira

**Affiliations:** 1 School of Public Health, Faculty of Medicine and Health, The University of Sydney, Sydney, New South Wales, Australia; 2 Institute for Musculoskeletal Health, School of Public Health, University of Sydney, Camperdown, New South Wales, Australia; 3 Institute for Musculoskeletal Health, Sydney Local Health District, Camperdown, New South Wales, Australia; 4 National University of Ireland Galway, Galway, Ireland; 5 Macquarie University, Sydney, New South Wales, Australia; 6 University of New South Wales, Neuroscience Research Australia, Sydney, New South Wales, Australia

**Keywords:** Musculoskeletal disorders, Back pain, Health Education

## Abstract

**Abstract:**

**Objectives:**

To explore how people perceive three different forms of advice for acute low back pain (LBP).

**Design:**

Content analysis of qualitative data collected in a three-arm randomised experiment.

**Participants:**

2200 participants with acute LBP (ie, pain duration for ≤6 weeks) were randomly assigned to receive three types of advice: guideline advice and guideline advice with the addition of either brief pain science or ergonomics messages.

**Primary and secondary outcomes:**

After receiving the advice, participants answered two questions: ‘If your health professional gave you this advice, how would it make you feel?’ and ‘If your health professional gave you this advice, what treatments (if any) do you think you would need?’ Two researchers coded responses using deductive content analysis.

**Results:**

We analysed 4400 free-text responses from 2200 participants. There were little to no differences in participants’ feelings, thoughts and expectations after receiving three types of advice for acute LBP. Participants most commonly expressed feeling positive about the advice (38%–35%), reassured (23%–22%) and empowered (10%–8%). Some expressed being unhappy or being frustrated with the advice (4%–3%). Participants most commonly thought they needed no treatment apart from staying active, followed by exercise and medication.

**Conclusions:**

Guideline advice with or without the addition of brief pain science or ergonomics messages generated positive feelings, reassurance or a sense of empowerment in many people with acute LBP, with no difference between types of advice.

**Trial registration number:**

ACTRN12623000364673.

**STRENGTHS AND LIMITATIONS OF THIS STUDY**

- We recruited a large sample of people with a lived experience of acute low back pain from five countries with different health systems.
- Two researchers independently double-coded all responses for each question while remaining masked to group allocation.
- We collected outcome data at one single point in time, immediately after participants were given the advice which may not reflect people’s long-term perceptions.
- Our sample had high health literacy which may limit the generalisability of findings to populations with low levels of health literacy.

## Introduction

 Low back pain (LBP) is a common health problem.^1^ The significant burden of LBP is evidenced by the large direct healthcare costs and indirect costs from lost productivity associated with it.^2 3^ For example, LBP is the primary reason reported for early retirement from the workforce.^4 5^ The prognosis of LBP is typically favourable, with most people recovering from an acute episode of LBP within 6 weeks, and therefore, guidelines recommend advice as first-line care to reassure people with LBP.^6^,^8^

Guidelines commonly recommend healthcare professionals to provide advice to people with acute LBP that explains the nature of LBP, its typically favourable prognosis, and also recommend that people are advised to stay active, avoid bed rest and remain at work. Two other approaches, advice based on pain science and ergonomics principles, are often added to guideline-based advice. Pain science aims to reframe unhelpful beliefs by providing information on the biological basis of pain and the protective purpose it serves.^9^ Messages based on ergonomics principles rely on a biomechanical framework to explain how postures and excessive loading of the spine (eg, lifting with incorrect technique, being overweight) can result in pain and disability. Advice based on ergonomics principles aims to educate people with acute LBP to reduce strain on the lumbar spine by maintaining good posture, practising safe lifting and/or reducing weight. The key contrast between advice based on ergonomic principles and guideline advice is the biomechanical framework used (in ergonomic advice) to explain how back pain develops and may persist. Using pain science messages to explain pain to patients has become a very popular approach among clinicians, whereas messages based on ergonomic principles are often used by the general public to make sense of LBP. Despite the popularity of these three types of advice, to date, there is very little evidence to inform clinicians on the best content to include when providing advice for acute LBP as previous systematic reviews had shown similar outcomes for these three types of advice.^10^

We recently completed a randomised experiment that showed that adding pain science messages to guideline advice did not provide more reassurance than guideline advice alone and that adding ergonomics advice provided worse reassurance compared with guideline advice alone.^11^ We also showed that people perceived guideline advice alone or advice combined with pain science or ergonomics messages to have similar levels of credibility. However, guideline advice alone was perceived as slightly less relevant in addressing participants’ concerns than guideline advice combined with ergonomics messages (but not pain science messages).

As part of that experiment, we collected qualitative data that could provide insights into people’s perceptions of the different types of advice and explain the findings from the experiment. The aim of this study was to explore participants’ feelings, thoughts and expectations and their perceived need for treatment evoked by three types of advice for acute LBP using qualitative data collected alongside a randomised experiment.

## Methods

### Study design

We used content analysis to analyse qualitative data collected in a three-arm parallel online randomised experiment.^11^ The study was registered in the ANZCTR (ACTRN12623000364673).

### Participants and recruitment

Recruitment and data collection took place from 4 April 2023 to 30 April 2023. We recruited participants with self-reported acute LBP who were 18 years or older from Australia, New Zealand, Canada, the UK and the USA. Participants were included in the study if they could read and type in English and they (1) self-identify as currently experiencing an episode of acute LBP (duration ≤6 weeks); (2) rate their LBP over the past week as ≥1 on a Numerical Pain Rating Scale of 0–10 (to exclude those who do not have LBP at the time of the intervention); (3) have not received a diagnosis of cancer, fracture in the lumbar spine, infection in the lumbar spine and cauda equina syndrome by a doctor and (4) have not undergone lumbar surgery.

Participants were recruited through Qualtrics, a market research company specialising in online panel survey research. Qualtrics partners with 20 panel providers globally to provide access to panels (demographics align with the national population) consisting of participants who have previously agreed to be involved in online survey research. Participation is incentivised using points which participants can redeem for cash, airline miles and gift cards. Qualtrics implements numerous strategies to avoid duplication (eg, IP address checks) and ensure the validity of responses (eg, Captcha verification to screen out bots, flagging speeders or suspicious open-ended responses).^12 13^

### Data collection

The questionnaire was adapted from previous published work.^12^,^16^ We piloted the questionnaire with 40 participants to test the suitability of the questionnaire. No changes were made after the piloting of the questionnaire. Data from this pilot testing are included in this study. Participants provided demographic information at the start of the questionnaire. This included data on age, sex, educational attainment, country of birth, employment status, private health insurance and health literacy using the single-item health literacy screener.^17^ Participants also provided information on their history of LBP and healthcare utilisation. This included duration of LBP (weeks), history of diagnostic investigations for LBP (X-ray, MRI or CT scan), current LBP pain level (measured using the 0–10 Numerical Pain Rating Scale, 0=no pain, 10=the worst pain), risk of developing chronic pain, measured using the PICK-UP risk assessment tool^18^ and emotional distress^19^ (‘how tense or anxious have you felt in the past week’ and ‘how bothered by feelings of depression have you been in the past week’ measured on a 0–10 scale). After participants provided demographic and clinical information, they were randomly assigned to one of the three groups; guideline-based advice, guideline-based advice plus pain science-based advice and guideline-based advice plus ergonomics-based advice (allocation ratio 1:1:1). The randomisation was completed using Qualtrics’ randomisation software, which uses the Mersenne Twister algorithm to generate the random allocation sequence.^20^ Participants then watched a video containing the key messages for each type of advice. Videos are available in online supplemental file 1.

Guideline advice messages were based on the Australian Commission on Safety and Quality in Health Care’s LBP clinical care standard.^6^ Key messages were to provide education about the nature of LBP, encourage continuing with normal activities and avoiding bed rest and encourage self-management. Pain science advice messages were adapted from the pain science education used in the PREVENT trial,^21^ providing pain science-based education compared with placebo. Ergonomics advice messages were drawn from the resources available for consumers on the HealthDirect website.^22^ HealthDirect is a health information website supported by the Australian Government. The advice emphasised the importance of maintaining good posture, practising safe lifting techniques and maintaining a healthy weight.

Outcome data were collected immediately after participants viewed the video. In this analysis, we focused on the free-text responses to the following two questions:

1. ‘If your health professional gave you this advice, how would it make you feel?’
2. ‘If your health professional gave you this advice, what treatments (if any) do you think you would need?’

### Data analysis

We used deductive^23 24^ content analysis to analyse the free-text responses to each question. Content analysis is an appropriate method for analysing text data as it uses both qualitative and quantitative methods and has been previously used successfully in similar studies.^15 25 26^ Content analysis allows the text to be classified into smaller categories (with text in the same category presumed to have similar meanings) and enables reporting of the frequency of categories.^25^ The analysis was an iterative process involving two researchers (LA and GEF).

We adapted an existing framework used in previous studies^12 15^ examining people’s thoughts, feelings and expectations around different disease labels and their influence on treatment intentions. Both studies used similar questions to examine the influence of disease labels rather than of advice. Two researchers independently reviewed all free-text responses to familiarise with the data, then coded the first 50 responses for each question using an initial framework containing predetermined categories with operational definitions and examples. The coding framework was exchanged to compare the codes for each response. Any disagreements between coders were resolved by consensus. Responses that were not coded using the initial framework were used to create new codes which were integrated to develop the coding framework. For example, the code ‘trust in expertise’ was used to capture any references to the advice/clinician being trustworthy. Participants who did not provide an answer to an open-ended question were excluded from the analysis of that question. Unusable and uninformative responses were coded as ‘irrelevant responses’ (eg, ‘I prefer not to answer’).

Using the modified coding framework, all free-text responses for each question were double-coded independently by two researchers (LA and GEF) to confirm consistency. All coding was performed blind to the allocation of groups. The analysis was an iterative process and reflects the judgements, practices and beliefs of a physiotherapist who has experience in qualitative research in musculoskeletal conditions and of a Master of Public Health student in qualitative research. Each response was coded using as many codes as appropriate. Members of the study team had the opportunity to comment and reflect on the results and their interpretation. Five was the highest number of codes allocated to a response. Where there was disagreement, notes were compared and disagreements between coders were discussed and resolved by consensus. We used Microsoft Excel V.16.54 to conduct our statistical analysis. Frequencies of codes were used to determine the prominence of themes. The coding framework for each question is provided in online supplemental file 2.

### Patient and public involvement

We sought advice from five consumers with LBP on their lived experience of having LBP and what concerned them. Their feedback was used to inform the development of the guideline-based messages intervention.

## Results

We recruited 2313 participants, of whom 93 did not answer the open-ended questions, thus 2220 (96%) were included in the analysis, totalling 4440 free-text responses. The participants in the three groups had similar characteristics (table 1). Participants were on average 38.4 years old and 58.2% were female. 21.2% (n=492) of participants reported no history of LBP; 19.1% (n=444) of participants reported a history of LBP and had received advice for LBP. The mean (SD) LBP intensity was 4.6.^2^ We coded 317 (14.3%) responses as irrelevant (eg, ‘prefer not to answer’). Participants spent a median (IQR) of 10 min (7.2–14.6) on the survey and watched the videos for an average of 88% (19.3) of their total duration.

**Table 1:** Baseline characteristics of participants (n=2200)

	Guideline advice (n=760)	Guideline advice+pain science advice (n=727)	Guideline advice+ergonomics advice (n=733)
Age (years), mean (SD)	38.6 (13.6)	38.2 (13.5)	38.8 (13.2)
Sex, n (%)			
Female	449 (59.1)	440 (60.5)	400 (54.6)
Male	310 (40.8)	286 (39.4)	331 (45.1)
Prefer not to say	1 (0.1)	1 (0.1)	2 (0.3)
Education			
Up to high school (not completed)	24 (3.2)	34 (4.7)	36 (4.9)
High school (completed)	172 (22.7)	162 (22.3)	140 (19.1)
Non-university tertiary education	92 (12.1)	113 (15.6)	126 (17.2)
University	471 (62.1)	416 (57.4)	431 (58.8)
Employment			
Employed	605 (79.6)	572 (78.7)	601 (82)
Unemployed	74 (9.7)	72 (9.9)	64 (8.7)
Student	24 (3.2)	32 (4.4)	14 (1.9)
Retired	57 (7.5)	51 (7)	54 (7.4)
Private health insurance (yes), n (%)	486 (64)	445 (61.2)	436 (59.5)
Health literacy (need help with medical instructions or materials), n (%)			
Never	373 (49.1)	349 (48)	365 (49.8)
Rarely	164 (21.6)	155 (21.3)	156 (21.3)
Sometimes	166 (21.8)	161 (22.1)	153 (20.9)
Often	47 (6.2)	52 (7.2)	50 (6.8)
Always	10 (1.3)	10 (1.4)	9 (1.2)
Previous low back pain (yes), n (%)	599 (78.8)	567 (78)	590 (80.5)
Received advice for LBP previously (yes), n (%)	459 (60.4)	434 (59.7)	437 (59.6)
Low back pain intensity (0–10), mean (SD) (range)	4.6 (2.1) (1–10)	4.7 (2.1) (1–10)	4.6 (2) (1–10)
Duration of current episode (weeks), n (%)			
<1 week	173 (22.8)	178 (24.5)	178 (24.3)
1–2 weeks	225 (29.6)	201 (27.7)	204 (27.8)
2–4 weeks	199 (26.2)	185 (25.5)	198 (27)
4–6 weeks	163 (21.5)	163 (22.4)	153 (20.9)
Leg pain (yes), n (%)	247 (32.5)	245 (33.7)	242 (33)
Low back pain interference, n (%)			
Not at all	83 (10.9)	98 (13.5)	86 (11.7)
A little	328 (43.2)	284 (39.1)	300 (40.9)
Moderately	242 (31.8)	237 (32.6)	240 (32.7)
Quite a bit	95 (12.5)	102 (14)	99 (13.5)
Extremely	12 (1.6)	6 (0.8)	8 (1.1)
Compensable episode* (yes), n (%)	160 (21.1)	159 (21.9)	144 (19.7)
Emotional distress (0–10), mean (SD)			
Anxiety symptoms (0–10)†	5.1 (2.3)	5.4 (2.3)	5.2 (2.2)
Depression symptoms (0–10)†	4.6 (2.8)	4.7 (2.8)	4.4 (2.8)

* For example, workers’ compensation.

† Higher scores indicate greater symptom severity.

LBPlow back pain

### Question 1: ‘if your health professional gave you this advice, how would it make you feel?’

30 themes emerged from the content analysis of responses to the three forms of advice. There were virtually no differences in the frequencies of themes observed between groups (table 2 and online supplemental file 3,4) so results are presented for the overall sample. The most common themes expressed across the types of advice were feeling positive about the advice (37.8%–34.6%), feeling reassured (23.4%–22%), feeling empowered (10.2%–7.8%), willingness to follow the advice (4.2%–3.1%), feelings of uncertainty (4.8%–3.2%), trust in the expertise of the physiotherapist delivering the advice (3.7%–3.1%) and feeling unhappy or frustrated with the advice (4%–3.1%).

**Table 2:** Top 10 themes from feelings, thoughts and expectations expressed by advice (n=2220)

Guideline-based advice (n=760)	Guideline-based advice+pain science-based advice (n=727)	Guideline-based advice+ergonomic-based advice (n=733)
Positive about the advice(n=263, 35%)	Positive about the advice(n=259, 36%)	Positive about the advice(n=277, 38%)
Reassured(n=178, 23%)	Reassured(n=160, 22%)	Reassured(n=170, 23%)
Empowered(n=59, 8%)	Empowered(n=67, 9%)	Empowered(n=75, 10%)
Willing to try/follow the advice(n=32, 4%)	Uncertainty(n=35, 5%)	Trust in expertise(n=24, 3%)
Unhappy/frustration(n=30, 4%)	Willing to try/follow the advice(n=29, 4%)	Uncertainty(n=24, 3%)
Trust in expertise(n=27, 4%)	Trust in expertise(n=27, 4%)	Willing to try/follow the advice(n=23, 3%)
Uncertainty(n=24, 3%)	Unhappy/frustration(n=19, 3%)	Unhappy/frustration(n=23, 3%)
Seek treatment/investigation(n=21, 3%)	Good prognosis(n=18, 2%)	Minor issue(n=21, 3%)
Less anxious/depressed(n=19, 3%)	Hopeful(n=17, 2%)	Good prognosis(n=20, 3%)
Good prognosis(n=17, 2%)	No impact on thoughts and/or feelings(n=16, 2%)	No impact on thoughts and/or feelings(n=15, 2%)

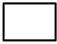
, <1%; 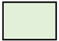, 1%–10%; 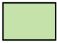, 11%–20%; 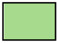, 21%–30%; 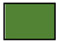, 31%–40%.

Those expressing being reassured by the advice often mentioned that the advice reassured them that their pain was not caused by a serious condition and that it was safe to move.

Good knowing that continuing to move and train my pain receptors to relax would most likely fix the pain. Optimistic and motivated.

[Female, age 32, received brief pain science messages added to guideline advice].

Feelings of uncertainty and unhappiness or frustration were often expressed when participants perceived the advice did not match their pain experience or when participants perceived that no importance was being placed on understanding what caused their LBP.

*A bit confused! It’s hard to carry on with most normal activities when you’re having a lot of pain. Especially when Dr says scan results are no further action required! Yet I’m still getting bad pain! I can’t keep taking panadeine forte* [NB: codeine + paracetamol]

[Female, age 75, received advice only]

*A bit frustrated as my back often gets so sore, that any movement causes me significant pain.* [Female, age 44 received brief ergonomics messages added to guideline advice]

### Question 2: ‘if your health professional gave you this advice, what treatments (if any) do you think you would need?’

31 treatments emerged from the content analysis (table 3 and online supplemental file 5,6). Across the three groups, participants most commonly thought they would need no treatment and that they needed to stay active (29.1%–27.3%), followed by exercise (17.7%–17.2%) and medication (12.5%–11.4%). Across the three groups, a similar percentage of participants reported being unsure about what treatments they would need based on the advice that they received (7.6%–6.6%) with no differences between groups.

**Table 3:** Top 10 treatments reported for each form of advice for LBP (n=2220)

Guideline-based advice(n=760)	Guideline-based advice+pain science-based advice (n=727)	Guideline-based advice+ergonomic-based advice (n=733)
No treatment/stay active(n=221, 29%)	No treatment/stay active(n=208, 29%)	No treatment/stay active(n=200, 27%)
Exercise(n=132, 17%	Exercise(n=125, 17%)	Exercise(n=130, 18%)
Medication(n=95%, 13%)	Rest(n=97, 13%)	Medication(n=89, 12%)
Rest(n=69, 9%)	Medication(n=83, 11%)	Rest(n=60, 8%)
Unsure(n=58, 8%)	Unsure(n=55, 8%)	Unsure(n=48, 7%)
Manual therapy(n=37, 5%)	Physiotherapy(n=31, 4%)	Physiotherapy(n=48, 7%)
Physiotherapy(n=37, 5%)	Manual therapy(n=29, 4%)	Manual therapy(n=40, 5%)
Imaging(n=29, 4%)	Imaging(n=28, 4%)	Posture correction(n=39, 5%)
Chiropractor(n=22, 3%)	Chiropractor(n=19, 3%)	Imaging(n=31, 4%)
Lifestyle changes(n=14, 2%)	Heat(n=18, 2%)	Lifestyle changes(n=27, 4%)

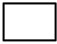
, <1%; 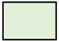, 1%–10%; 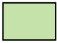, 11%–20%; 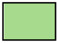, 21%–30%.

LBPlow back pain

Overall, participants more often thought that they would need non-surgical treatments such as physiotherapy (6.6%–4.3%) and manual therapy (5.5%–4%) than invasive treatments such as surgery (1.8%–1.6%) and injections (0.1%).

Rest was most often expressed by participants receiving guideline advice only (13.4%) and least expressed by participants who received ergonomics messages (8.2%). The need for postural correction emerged as a treatment more commonly among those who received ergonomics messages (5.3%) and less commonly among those receiving guideline advice only (0.4%) and pain science messages (0.4%). There were no other marked differences in the frequencies of treatement themes expressed across the types of advice (online supplemental file 4).

A few participants expressed that the severity of their LBP was an important factor driving their desire for receiving more information about potential treatment options.

*If my level of back pain was more severe I would like to hear more options of what it could be however I think the advice given listed what my current back pain situation is very well*.

[Female, age 25, received brief pain science messages added to guideline advice]

## Discussion

### Summary of key findings

Our analysis found little to no difference in participants’ feelings, thoughts and expectations after receiving three types of advice for LBP that varied with regard to the messages conveyed. Overall, participants receiving guideline advice only or guideline advice combined with pain science or ergonomics messages felt positive, reassured and empowered regardless of the type of advice that they received. They less commonly expressed feelings of uncertainty or unhappiness and frustration towards the advice received. After receiving each form of advice, participants most commonly thought they needed no treatment apart from staying active, followed by exercise and medication. Non-surgical treatments such as physiotherapy were more commonly mentioned than invasive treatments such as surgery.

### Strengths and limitations of this study

This study has several strengths. We recruited a large sample of people with a lived experience of acute LBP recruited from five countries with different health systems. There was a high response rate for both questions. Two researchers independently double-coded all responses for each question while remaining masked to group allocation.

Our study has limitations. Our experiment was conducted online, and findings may differ had the experiment been conducted in patients seeking care in the real world. We collected outcome data at one point in time, immediately after participants were given the advice. Our findings may have been different as participants reflected further over time. Advice in our study was delivered by a physiotherapist via brief prerecorded videos. Our findings may have been different if the advice had been delivered by a health professional (eg, general practitioner, physiotherapist) during a clinical encounter. We did not include non-English-speaking participants, which limits the generalisability of findings to linguistically diverse populations. Our sample was overall well educated and had high health literacy. We did not explore participants’ previous experiences in depth, so we do not know whether the intervention changed participants’ pre-existing thoughts and feelings about their LBP. We also did not explore whether participants understood the messages that were delivered to them in each group.

### Comparison with previous studies

In this content analysis, we found little to no difference in the proportion of participants expressing being reassured across the three groups. This aligns with the findings from the quantitative analysis of this randomised experiment, which found that adding pain science or ergonomics messages to guideline advice did not increase reassurance or change management intentions in people with acute LBP.^11^ The quantitative analysis also found that ergonomics messages may lead to reduced feelings of reassurance, however, our content analysis was not able to identify thoughts and feelings that might help explain that finding.^11^

The most commonly expressed emotions and feelings after advice in our content analysis were positive (eg, reassurance, empowerment). These findings are similar to those from a recent randomised experiment in people with shoulder pain comparing guideline-based advice with structural-based advice, which found that participants receiving guideline-based advice more often expressed feelings of reassurance and having a minor issue compared with participants receiving structural-based advice.^14 26^ A difference from that trial is that we did not observe marked differences in evoked feelings between the different types of advice. One potential explanation is that while that study provided two contrasting forms of advice, our experiment investigated the effect of adding pain science and ergonomics messages to guideline advice. Key aspects of guideline advice for acute LBP (eg, advice to stay active, avoid bed rest, benefits of regular physical activity) are typically provided alongside pain science and ergonomics advice,^21^ so attempts to create advice using pain science and ergonomics messages that did not contain any element of guideline advice would not have been representative of how those messages are conveyed to patients.

### Meaning of the study

While many expressed being reassured after the advice and there were no marked differences in the thoughts and feelings evoked by each type of advice separately, our content analysis identified a large range of themes that can offer insights into key individual attitudes and beliefs towards advice for acute LBP and inform future work. For example, some perceived the advice as dismissive, which most commonly happened when the advice did not match their pain experience or when participants perceived that no importance was being placed on understanding what caused their LBP. Previous work has shown that people with LBP want clear information about diagnosis,^27^ and that people with LBP consider information on the identification of serious causes of LBP as the most important key priority messages.^28^ Our findings add to a body of evidence suggesting that more work is needed to craft advice messages that are more relevant to people with LBP.

Adding pain science or ergonomics messages to guideline-based advice had little to no impact on participants’ perceptions of the advice provided. There were only two themes where a noticeable (although small) difference between groups was observed: rest and postural correction. Rest was more often expressed as a required treatment to those in the guideline advice-only group, and least expressed in the group that received ergonomics messages, whereas postural correction was more commonly expressed in the group that received ergonomics messages compared with the other two types of advice. The proportion of participants in our study who thought rest was an appropriate treatment for their LBP is similar to that reported by previous studies. For example, Hall *et al* found that 6% of participants with LBP reported resting in bed to manage their pain.^29^ Postural advice is postulated to be harmful, and our randomised experiment did find some evidence that ergonomics messages reduced reassurance that it was safe to move. Our content analysis offers some insights on factors that may potentially mediate a relationship between ergonomics advice and reduced reassurance, such as the role of the belief that postural correction is a required treatment.

Our content analysis found that about one-third of participants thought they did not need any treatment after receiving either form of advice. Whether that would be translated to a reduction in care-seeking behaviour in real life is unknown, as the effect of advice on care seeking has been little explored. For example, there is limited evidence that receiving intensive pain science education (compared with placebo pain science education) may reduce care seeking. In the PREVENT trial,^21^ those receiving pain science education were 43% less likely to seek care in the first 3 months after the intervention (but not at 6 and 12 months) than those randomised to placebo pain science education. Advice and education when provided as part of mass media campaigns have had mixed results in terms of reducing care seeking for LBP.^30^ Given the high prevalence of care seeking related to acute LBP,^31^ future trials could investigate the effectiveness and cost-effectiveness of interventions such as guideline advice in reducing care seeking for LBP in different settings (eg, as part of a mass media campaign using social media or at the point of care for people with acute LBP).^32^ Results from our content analysis combined with the findings from the quantitative analysis indicate that adding pain science or ergonomics messages to campaigns targeted to reduce care seeking may not be needed.

## Conclusion

Guideline advice with or without pain science or ergonomics messages generated positive feelings, reassurance or a sense of empowerment in many people with acute LBP, with no difference between types of advice. However, for some, the advice generated uncertainty or was perceived as dismissive of their condition. Our findings add to a body of evidence suggesting that more work is needed to craft advice messages that improve outcomes for people with acute LBP.

## supplementary material

10.1136/bmjopen-2023-079070 online supplemental file 1

## Data Availability

Data are available on reasonable request.
