# Supplementary material for: Perceptions of advice for acute low back pain: a content analysis of qualitative data collected in a randomised experiment
Source: BMJ Open. 2024 Jul 23;14(7):e079070. doi: 10.1136/bmjopen-2023-079070 (PMC11268038; doi:10.1136/bmjopen-2023-079070)
Supplement: online supplemental file 1 [file bmjopen-14-7-s001.pdf]

Supplementary file 1. Videos.

Guideline advice

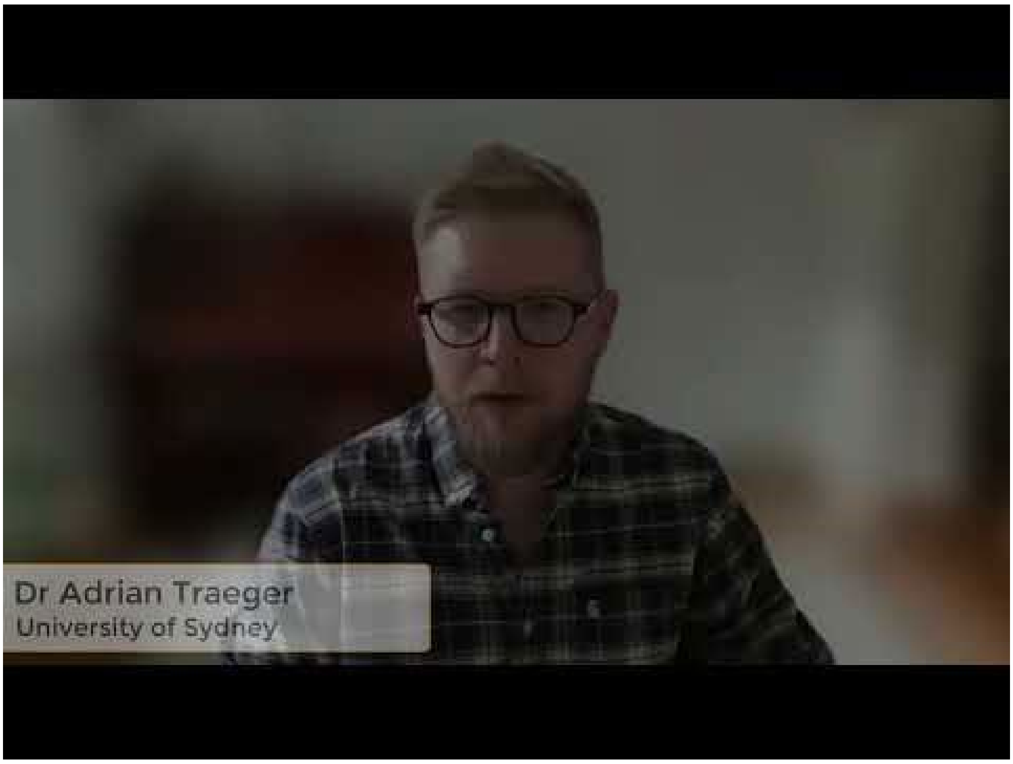

Guideline advice + pain science messages

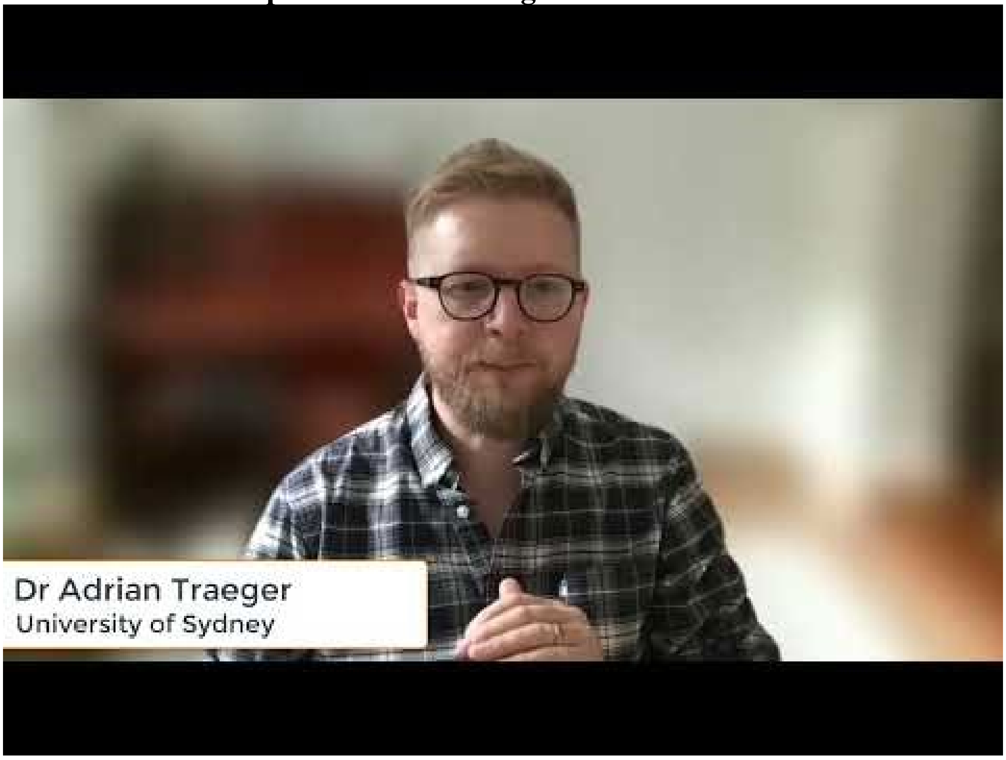

Guideline advice + ergonomics messages

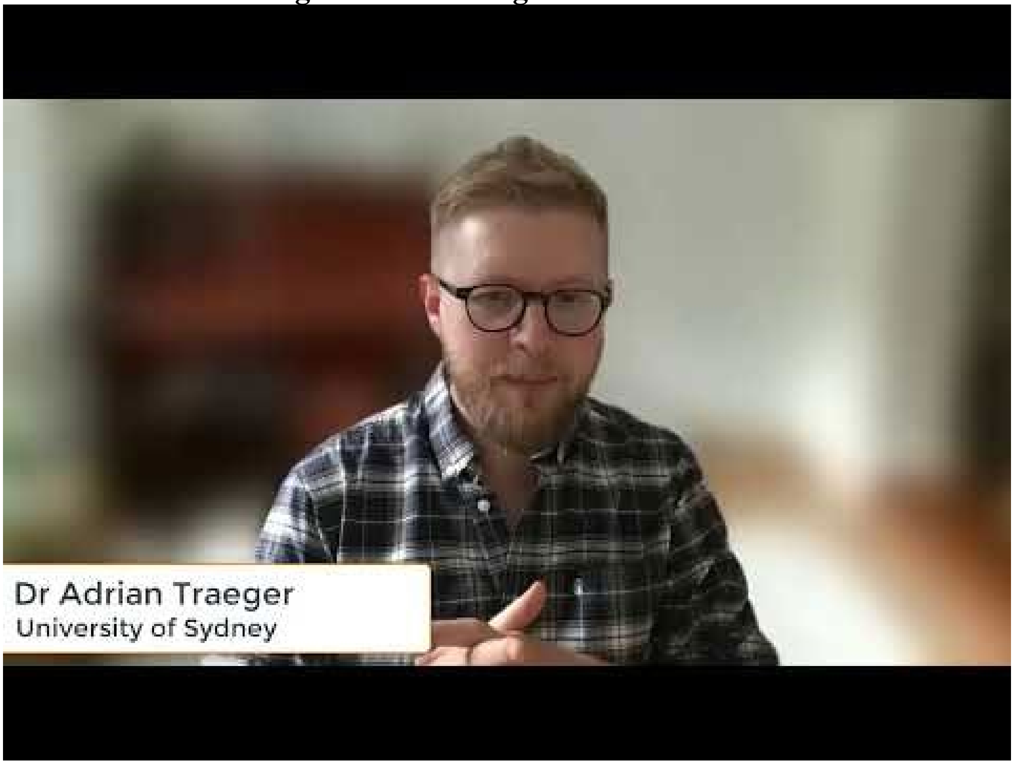

Supplementary File 2. Coding frameworks

Question 1: If your health professional gave you this advice, how would it make you feel?

**Code**	**Explanation**	**Examples**
Activity restriction	Any reference to being unable to do typical daily activities	Caution, light work, rest, sleep loss, time off work, careful
Aging	Any reference to the condition being due to aging	Old, getting old/older, ancient
Avoid treatment/investigation	Any reference the need/willingness to avoid treatment or investigation	Avoid seeing doctors, avoid (unnecessary) scans, medicines
Challenged preconceptions	Any reference to feeling challenged on previous attitudes or beliefs	Surprised rest is not needed, new advice
Empowered	Any reference to improved capacity to manage the condition/knowing what to do to get better	Confident, informed, knowledge
Feels dismissed	Any reference to feeling dismissed by another person	Not interested in my opinion, not bad to those who don't suffer from it, not real, made up
Good prognosis	Any reference to the condition recovering either quickly or without treatment	Temporary, no treatment needed, heal over time
Have received similar advice before	Any reference to previous advice that is concordant to the advice received in the study	Received the exact same advice from the doctor
Hopeful	Reference to there being things that can be done to help	Optimistic
Irrelevant response	The response did not address the question	"Nothing at all", "I don't really have any feelings", as per professionals' advice
Less anxious/depressed	Any reference to feeling less anxious or depressed	Relaxed, psychological relief
Mechanism of injury	Any reference to why the pain started	Injury, overuse issue, caused by lifting, sports injury [it talks about cause and how knowing

		what's causing it was seen as good news]
Minor issue	Any reference to the condition being ‘non-serious’	Not serious, everyday issue, common, annoyance, uncomfortable, inconvenient
No impact on thoughts and/or feelings	Any reference to advice having no impact on thoughts or feelings	Neutral, feels no different
Pain experience	Any reference to pain	Pain, hurt, intermittent, discomfort, recurrent
Poor prognosis	Any reference to the condition taking a long time to recover	Persistent pain, long recovery, long-term issue
Positive about the advice (not captured by other codes)	Any reference to positive emotions that are not captured by other codes	Good, comfortable
Psychological distress	Any reference to feelings of fear, anxiety, worry or stress	Fear, anxious, worry, stress, scared, depressed, nervous, etc.
Reassured	Any reference to the advice being reassuring	Reassured back pain isn’t serious; happy it’s not a serious disease; glad I have nothing, relief, comforted, relaxed, at ease, not worried, less concerned.
Remain active	Any reference to thinking about being active	Continue to move, regular exercise
Seek treatment/ investigation	Any reference to the need for treatment or investigation	Rest, pain medication, heat, surgery, physiotherapy, requires imaging
Serious issue	Any reference to the condition being ‘serious’	Deteriorating, serious, bad, very ill
Tissue damage or dysfunction	Any reference to tissue damage or dysfunction	Tendon tear, arm out of place, sprained ligaments, pulled muscle, stiffness, weakness
Trust in expertise	Any reference to trusting because advice/clinician is being trustworthy	Reliable, credible

Uncertainty	Any reference to being unsure what the advice means	Complicated, confused, uncertainty, need more information, seeking second opinion, try and see if it works
Understanding of the cause of pain	Any reference to improved understanding of the cause their pain	Feels more knowledgeable, reasons for the cause of pain
Unhappy/ frustration	Any reference to being unhappy or frustrated	Sad, anger, annoyed, feel bad, upset, helpless, useless
Usefulness dependent on the severity	Any reference to the usefulness of the advice conditioned on the intensity of the pain	Accepting of the advice because condition is minor.
Valued/heard	Any reference to feeling valued or cared for	Understood, caring, empathetic
Willing to try/follow the advice	Any reference to willingness to follow the advice	Would try to follow the advice
Would like tailored advice	Any reference to wanting advice specific to one's condition.	A bit vague, not specific to the problem

Question 2: If your health professional gave you this advice, what treatments (if any) do you think you would need?

**Code**	**Examples (if needed)**
Activity modification	Avoid lifting, avoid aggravating activities, avoid strenuous activities
Acupuncture	Dry needling
Chiropractor	Chiropractic adjustment
Cold	Cold packs, ice gel
Diet	Tonic food, diet therapy
Doctor	"I need a health check-up"
Electrotherapy	Laser, ultrasound
Emergency department/hospital	Require immediate medical attention
Ergonomics/posture	Adjust computer screen height
Exercise	Yoga, Pilates
Good mattress	Better bed

Heat	Heat pack, hot compress, hot showers
Hormones	Hormonal balance
Immobilisation	Sling
Injection	Cortisone injection
Investigations	X-ray, ultrasound, MRI
Irrelevant response	As per the advice
Light exercise	Gentle exercise, exercise but be careful
Manipulation	Alignment, adjustments
Massage	Deep massager, remedial massages
Medication	Panadol, anti-inflammatories, muscle relaxants, supplements
Natural or unknown therapies	Stone therapy, finger therapy, natural remedies, tea, spa baths, moxibustion
No treatment	Time, patience, will heal itself in time
Normal movements	Keep arm moving, normal activity, stay active, stay more active
Osteopathy	Osteopath, Osteotherapy
Other treatments	clinical treatments, lumbar treatment
Pain clinic	Pain management
Physiotherapy	Physical therapy, rehabilitation
Psychological therapy	Cognitive behavioural therapy
Rest	Taking it easy, relaxation, reduce overall activity
Second opinion	Need to find an expert, alternative advice to confirm
Self-management	At home
Specialist	Need to see a specialist
Stay healthy	Good sleep, avoid smoking, reduce stress
Stretching	Stretching exercises, stretches to loosen the nerve
Surgery	Disc replacement, minimally invasive surgery
Taping/bracing	Brace, strapping
Time off work	A day off at home, stay away from work for while
Topical treatments	Ointment, rub, Voltaren gel, oils, plaster
Traction	
Unsure	Not sure what treatment is needed, need to discuss further
Wait and see	Monitor pain, no treatment unless pain got worse
Weight loss	

Supplementary File 3. Themes from feelings, thoughts, and expectation evoked by the three forms of advice ranked from most to least common in each group.

Total sample (n= 2,220)	Guideline-based advice (n=760)	Guideline-based advice + pain science-based advice (n=727)	Guideline-based advice + ergonomic-based advice (n=733)
Positive about the advice (n=799, 36%)	Positive about the advice (n=263, 35%)	Positive about the advice (n=259, 36%)	Positive about the advice (n=277, 38%)
Reassured (n=508, 23%)	Reassured (n=178, 23%)	Reassured (n=160, 22%)	Reassured (n=170, 23%)
Empowered (n=201, 9%)	Empowered (n=59, 8%)	Empowered (n=67, 9%)	Empowered (n=75, 10%)
Willing to try/follow the advice (n=84, 4%)	Willing to try/follow the advice (n=32, 4%)	Uncertainty (n=35, 5%)	Trust in expertise (n=24, 3%)
Uncertainty (n=83, 4%)	Unhappy/frustration (n=30, 4%)	Willing to try/follow the advice (n=29, 4%)	Uncertainty (n=24, 3%)
Trust in expertise (n=78, 4%)	Trust in expertise (n=27, 4%)	Trust in expertise (n=27, 4%)	Willing to try/follow the advice (n=23, 3%)
Unhappy/frustration (n=72, 3%)	Uncertainty (n=24, 3%)	Unhappy/frustration (n=19, 3%)	Unhappy/frustration (n=23, 3%)
Good prognosis (n=55, 2%)	Seek treatment/investigation (n=21, 3%)	Good prognosis (n=18, 2%)	Minor issue (n=21, 3%)
Minor issue (n= 51, 2%)	Less anxious/depressed (n=19, 3%)	Hopeful (n=17, 2%)	Good prognosis (n=20, 3%)
Less anxious/depressed (n= 44, 2%)	Good prognosis (n=17, 2%)	No impact on thoughts and/or feelings (n=16, 2%)	No impact on thoughts and/or feelings (n=15, 2%)
No impact on thoughts and/or feelings (n=43, 2%)	Minor issue (n=15, 2%)	Minor issue (n=15, 2%)	Less anxious/depressed (n=14, 2%)
Hopeful (n=40, 2%)	No impact on thoughts and/or feelings (n=12, 2%)	Seek treatment/investigation (n=11, 2%)	Hopeful (n=13, 2%)
Seek treatment/investigation (n=37, 2%)	Hopeful (n=10, 1%)	Less anxious/depressed (n=11, 2%)	Valued/heard (n=11, 2%)
Valued/heard (n=24, 1%)	Would like tailored advice (n=10, 1%)	Psychological distress (n=9, 1%)	Would like tailored advice (n=10, 1%)
Would like tailored advice (n=24, 1%)	Pain experience (n=9, 1%)	Valued/heard (n=7, 0.96%)	Have received similar advice before (n=7, 0.95%)
Psychological distress (n=21, 0.95%)	Psychological distress (n=8, 1%)	Activity restriction (n=7, 0.96%)	Seek treatment/investigation (n=5, 0.68%)
Pain experience (n=17, 0.77%)	Have received similar advice before (n=7, 0.92%)	Feels dismissed (n=6, 0.83%)	Feels dismissed (n=5, 0.68%)
Have received similar advice before (n= 17, 0.77%)	Valued/heard (n=6, 0.79%)	Pain experience (n=5, 0.69%)	Psychological distress (n=4, 0.55%)

Feels dismissed (n= 16, 0.72%)	Feels dismissed (n=5, 0.66%)	Would like tailored advice (n=4, 0.55%)	Understanding of the cause of pain (n=6, 0.82%)
Activity restriction (n=14, 0.63%)	Usefulness dependent on the severity (n=5, 0.66%)	Serious issue (n=4, 0.55%)	Activity restriction (n=4, 0.55%)
Understanding of the cause of pain (n=12, 0.54%)	Remain active (n=4, 0.53%)	Have received similar advice before (n=3, 0.41%)	Remain active (n=4, 0.55%)
Remain active (n=11, 0.50%)	Serious issue (n=4, 0.53%)	Remain active (n=3, 0.41%)	Pain experience (n=3, 0.41%)
Serious issue (n=10, 0.45%)	Mechanism of injury (n=4, 0.53%)	Mechanism of injury (n=3, 0.41%)	Avoid treatment/investigation (n=3, 0.41%)
Mechanism of injury (n=9, 0.41%)	Challenged preconceptions (n=4, 0.53%)	Understanding of the cause of pain (n=3, 0.41%)	Serious issue (n=2, 0.27%)
Usefulness dependent on the severity (n=8, 0.36%)	Activity restriction (n=3, 0.39%)	Usefulness dependent on the severity (n=2, 0.28%)	Mechanism of injury (n=2, 0.27%)
Poor prognosis (n=5, 0.23%)	Understanding of the cause of pain (n=3, 0.39%)	Poor prognosis (n=2, 0.28%)	Usefulness dependent on the severity (n=1, 0.14%)
Challenged preconceptions (n=5, 0.23%)	Poor prognosis (n=2, 0.26%)	Challenged preconceptions (n=1, 0.14%)	Poor prognosis (n=1, 0.14%)
Avoid treatment/investigation (n=4, 0.18%)	Avoid treatment/investigation (n=1, 0.13%)	Aging (n=1, 0.14%)	Challenged preconceptions (n=0, 0.00%)
Aging (n=1, 0.05%)	Aging (n=0, 0.00%)	Avoid treatment/investigation (n=0, 0.00%)	Aging (n=0, 0.00%)

<1%	1% - 10%	11% - 20%	21% - 30%	31% - 40%

Supplementary file 4. Illustrative quote for question 1.

Themes	Guideline-based advice	Guideline-based advice + pain science-based advice	Guideline-based advice + ergonomic-based advice
Activity restriction	*"cautious"* [P86, Male, age 55]<br>*"A bit unsure because I usually rest my back, not carry on as normal"* [P383, Female, age 33]	*"I would be more alert with what I can and can't do regarding my back"* [P1044, Male, age 37]<br>*"I'm afraid I won't be able to work in the future"* [P669, Female, age 33]	*"I would feel relieved that it isn't something serious and is something I can help manage and prevent at home with exercise and safety precautions and by using equipment to help reduce pressure on my back"* [P1789, Female, age 26]
Aging	N/A	*"Very reassured, because my biggest worry is the back pain becoming worse with ageing."* [P599, Male, age 37]	N/A
Psychological distress	*"I feel overwhelmed"* [P343, Female, age 60]	*"A bit concerned"* [P2073, Male, age 52]<br>*"It would be reassuring that it was nothing long term damage but I would potentially be anxious still just because of my mindset"* [P1240, Female, age 30]	*"Fear of illness."* [P1356, Male, age 36]
Feels dismissed	*"Dismissed. I have been diagnosed with arthritis that has been worsening. It's already forced one hip replacement. For someone to say all I need to do is move about more seems dismissive, at best."* [P789, Female, age 64]<br>*"That he was minimizing my concerns."* [P2013, Male, age 58]	*"Dismissed"* [P1819, Female, age 47]<br>*"Like he's looking for an easy way out"* [P374, Male, age 39]	*"Dismissed, and not taken seriously"* [P149, Female, age 28]<br>*"Like he was generalizing without conducting an actual exam"* [P730, Female, age 54]
Good prognosis	*"I would feel very confident that my back pain would be cured."* [P1699, Female, age 27]<br>*"more confident and sure that I will improve"* [P2208, Female, age 46]	*"Confident that the pain will go away eventually."* [P2127, Male, age 37]<br>*"Confident the pain would go away in time"* [P444, Male, age 79]	*"A lot better after being reassured it's not a long-term issue"* [P2012, Female, age 34]<br>*"It would be good, although I'm very big on wanting an immediate fix, though I know in this instance it will take time and that is necessary"* [P1076, Female, age 26]
Mechanism of injury	*"Relieved that it was just the ligaments and joints and nothing serious."* [P2066, Female, age 46]	*"It would make me feel confident that I am fine. That it's muscular."* [P1421, Male, age 58]<br>*"Somewhat relieved, but I still worry about scoliosis, or*	*"Ok, but not relevant to me as my back pain is just from the gym"* [P524, Male, age 22]<br>*"Good advice however not relevant to back pain from cramps"* [P34, Female, age 31]

		degenerating discs." [P853, Female, age 64]	
Minor issue	*"I feel that my disease is not very serious, I can still go to work normally, and it does not affect my life too much."* [P1649, Female, 37] *"Confident that it wasn't cancer, and that's it's normal/common"* [P780, Female, age 41]	*"More confident that the pain doesn't indicate a damage in muscles."* [P1328, Male, age 34] *"I would feel reassured that is not too serious or life threatening."* [P855, Female, age 23]	*"I feel better knowing that back pain is such a common occurrence with many people and that there are many different things that could be the cause of pain"* [P601, Female, age 70] *"Relieved to know that things are not as serious as I thought and happy to know there are simple solutions"* [P172, Male, age 60]
Pain experience	*"Not reassured because I already workout and it does not help with my back pain"* [P250, Female, age 22]	*"Good knowing that continuing to move and train my pain receptors to relax would most likely fix the pain. Optimistic and motivated."* [P947, Female, age 32] *"It would lessen my anxiety about back pain for sure. However, I'm not 100% sure how knowing this will actually reduce pain - just because I understand the pain now, doesn't mean it will disappear."* [P1665, Female, age 32]	*"A bit frustrated as my back often gets so sore, that any movement causes me significant pain."* [P1988, Female, age 44]
Poor prognosis	*"Make me feel ok but I think there would still be some concerns about the long term"* [P990, Male, age 23]	*"I'd listen and try it. If no improvement over time, I'd get a scan or try a chiropractor."* [P725, Female, age 63]	*"I'm afraid I won't be able to work in the future"* [P670, Female, age 32]
Reassured	*"Reassured that my back pain is not going to be a long-term condition and doesn't need any treatment"* [P1462, Female, age 34] *"Reassured that I can continue normal activity."* [P1057, Female, age 35] *"I think I would feel reassured that moving isn't going to make my back pain worse"* [P483, Female, age 56].	*"Relieved that my back pain isn't serious indicator of a worse condition."* [P954, Female, age 23] *"It would make me feel reassured and relived that it's most likely nothing serious and is very treatable."* [P513, Female, age 31]	*"It would reassure me that nothing is wrong with my back, and I would follow all the instructions."* [P983, Male, age 38] *"It would give me guidelines as to what to do and what not to do and reassure me"* [P99, Male, age 71]
Serious issue	*"It would make me feel a lot more at ease, I have had a bit of worry about my back in regard to it being an underlying serious issue as it has been lingering on and off for a few months. It has been*	*"Fine if they had ruled out any more serious cause"* [P76, Female, age 54]	*"I think its a compounding issue. The back pain itself is part of a bigger problem and I think I'd then need advice on weight loss."* [P1112, Female, age 30]

	coming and going" [P250, Female, age 22] *"Worry that lower back pain can lead to osteoporosis."* [P1466, Male, age 37]		
Seek treatment/ investigation	*"It would make me feel relieved however I would probably want some further validation with physio treatment or a scan"* [P939, Female, age 28] *"Nervous and I would most likely book in to get it checked properly with scans etc"* [P927, Female, age 35]	*"I'd have relief because I'm able to seek for the answers I needed, having enough rest will help that and seeking help from a specialist."* [P354, Female, age 24] *"I would want to have another ct scan done to make sure there are not any other underlying problems"* [P1538, Female, age 51]	*"If the cost of treatment is within my ability, I will go for treatment"* [P1090, Male, age 40] *"Thank you very much for the expert's advice, I will follow the expert advice to do, I will also combine work and rest, I believe that my body will be more and more healthy."* [P1730, Male, age 40]
Avoid treatment/investigation	*"I'd feel good that they're not immediately jumping to drugs or surgery"* [P2094, Male, age 61]	N/A	*"A whole lot better. It is the first time I have heard a solution outside of pain meds."* [P1792, Male, age 47] *"I would feel well informed. Instead of suggesting a bunch of tests it would make me feel like the care and want to explain things to me rather than just prescribe solutions."* [P2006, Male, age 32]
Uncertainty	*"I would find another health professional to help me."* [P288, Male, age 73] *"A bit confused! It's hard to carry on with most normal activities when your having a lot of pain. Especially when Dr says scan results are no further action required! Yet I'm still getting bad pain! I can't keep taking panadeine forte"* [P2046, Female, age 75]	*"Some comfort but I think I would still have some doubts."* [P1958, Female, age 31] *"it would make me feel a little sceptical"* [P586, Male, age 52] *"It would make me ask for a second opinion"* [P245, Female, age 56]	*"Happy with it, but the nice thing about visiting a healthcare professional is their ability to diagnose from an un-biased 3rd party perspective. For example, the video offered many possible reasons for the back pain and more than one apply but I am unsure to what extent each individual reason applies."* [P549, Male, age 23] *"Pretty good but would need more information"* [P2081, Female, age 29] *"Reassured but with lingering questions"* [P1971, Male, age 26]
Unhappy/ frustration	*"The health professionals keep giving the same information but don't bother finding out the problem."* [P946, Female, age 30] *"Like he was patronizing me, that he knew nothing, and " where will I try next?"* [P1747, Male, age 76]	*"Like I wasted my time seeing them."* [P487, Female, age 33] *"Not really related to the cause of my current pain."* [P2106, Female, age 24] *"Due to the reasons for my lower back pain, this would not help me at all."* [P841, Female, age 73]	*"I would question it ... when I try to resume normal daily active I often end up prolonging recovery ... that has always been the same for me"* [P20, Female, age 72] *"Misunderstood"* [P132, Female, age 42]

	"I've gotten this advice before. Hard to go about life while resting my back" [P527, Female, age 57]		*"I would feel like the advice is too generic"* [P863, Male, age 28]
No impact on thoughts and/or feelings	*"I already know all of this so the advice was nothing new"* [P1635, Female, age 46] *"Same as I do now"* [P81, Female, age 52]	N/A	*"I'm not sure I would have any specific feelings about this"* [P355, Female, age 45] *"No strong feelings either way"* [P685, Female, age 52]
Hopeful	*"I would feel optimistic."* [P117, Female, age 36] *"I would feel reassured and optimistic about improving my back pain"* [P508, Female, age 28]	*"It would make me feel like I had an option"* [P728, Female, age 43] *"It would be reassuring. I would assuage some fears. It would give me some idea and a plan to look forward to and to be slightly optimistic."* [P1744, Male, age 38]	*"more optimistic that I will get better by doing fairly simple things."* [P583, Male, age 58]
Empowered	*"Feels empowered to deal with the pain myself."* [P381, Male, age 45] *"Much better and in control"* [P883, Female, age 70]	*"Empowered to make better decisions for myself without worrying too much about it being serious. Great to know what pain is"* [P1533, Female, age 24] *"Confident and reassured that it's nothing serious and that I can do things to help improve/manage the pain."* [P1954, Female, age 29]	*"The advice I receive from a health professional would make me feel very confident in my everyday life. I would be reassured that I could perform tasks confidently if I followed a "never stop moving" regime."* [P45, Female, age 73] *"That I could do some things to address my pain without medical intervention"* [P1326, Female, age 55]
Trust	*"I would trust the advice given to me by experts"* [P1256, Male, age 31] *"Well I would definitely feel that they know what they are doing."* [P814, Male, age 30]	*"I would trust the health professional"* [P432, Female, age 43] *"I will follow his advice and it feels very credible."* [P1786, Female, age 37]	*"I would trust this advice very much"* [P1653, Female, age 34] *"It would make me feel confident that they are giving me the right advice"* [P277, Female, age 38]
Would like tailored advice	*"It would feel like good advice but a bit generic as though they don't have any specific advice"* [P972, Female, age 32] *"It's a bit vague and not specific to my problem, but it's sound advice"* [P2045, Female, age 24] *"I would want advice tailored to me, but it is helpful information"* [P56, Female, age 24]	*"It may be true for most people, but I have kidney and heart issues."* [P455, Female, age 50] *"This is made by doctors according to the actual condition of patients, without specific recommendations and indicators,"* [P1179, Female, age 38]	*"Comforted somewhat - advice specific to me would make me more confident i.e. physical exercises I could do to train the muscles/ligaments that reduce back pain"* [P919, Female, age 30] *"It's very general advice without actually knowing what sort of back pain the patient is suffering from so if a doctor told me this without investigating what the problem was I would think they haven't done their job*

			properly" [P62, Female, age 42]<br>*"I have been given this advice - and I applied it. I felt reassured. I just had knee replacement surgery - so not as active - and does affect the back."* [P2149, Female, age 61]
Valued/heard	*"I'd feel heard and respected for my problems not being deflected into a different matter"* [P1910, Female, age 22]<br>*"Listened to and valued*" [P1095, Female, age 33]	*"I will feel very appreciated and valued."* [P1754, Female, age 37]<br>*"He understands what I need"* [P522, Female, age 68]	"*Heard and actually listened to"* [P1796, Female, age 21]<br>*"I would feel reassured, and I would try what they suggested. I would feel like they listened to me and what the issue was."* [P238, Female, age 32]
Less anxious/depressed	*"Less anxious about it being serious"* [P1063, Female, age 23]<br>*"I'll feel a lot better, less depressed, more active therapy"* [P1854, Male, age 43]	*"If my expert could give me this advice, I would feel less anxious and know how to relieve pain and improve it"* [P741, Male, age 38]<br>*"I would definitely feel less stressed."* [P698, Female, age 31]	*"Makes me feel less anxious about my lower back pain"* [P209, Male, age 39]<br>*"I'll feel a lot better, less depressed, more active therapy"* [P1853, Male, age 43]
Willing to try/follow the advice	*"I would listen and heed what they have to say"* [P1560, Female, age 71]<br>*"I would be encouraged to try it."* [P1651, Male, age 73]	*"I would accept the information"* [P803, Female, age 39]<br>*"It would reassure me that there's a solution, and I would make the effort to follow the advice."* [P859, Female, age 54]	*"Feel fine taking on this advice"* [P970, Female, age 32]
Usefulness dependent on the severity	*"If it was excruciating pain that is further triggered while working I would not be happy with this advice. But it's acceptable as I only have mild soreness"* [P441, Male, age 23]<br>*"If it matches my symptoms, I would like to give it a try"* [P1087, Male, age 38]<br>*"If I had severe back pain it wouldn't make me feel good if you have and injured back you don't add exercise to it and add more damage"* [P2002, Female, age 70]	*"Reassured that nothing is wrong. I think of my pain was worse, I'd feel the opposite though, as if I wasn't being listened to"* [P1302, Female, age 27]<br>*"It would be reassuring. If my level of back pain was more severe I would like to hear more options of what it could be however I think the advice given listed what my current back pain situation is very well."* [P2086, Female, age 25]	*"It would put my mind at ease that it's nothing something very serious. Because it's only mild pain and the advice given makes sense, since I do work at a desk for long periods of time."* [P540, Female, age 37]
Positive about the advice (not captured by other codes)	*"It makes me feel comfortable and better"* [P283, Female, age 40]	*"This advice was very helpful, and I would feel great if my doctor with give me this advice"* [P823, Female, age 27]	*"I would think it was good and feel better because they were very logical reasons that made sense to me"* [P743, Female, age 20]

	"I would feel happy with the information provided" [P1955, Female, age 47]		
Have received similar advice before	*"Same as already received from my doctor"* [P148, Male, age 64]<br>*"Much more comfortable and I have had this advice before by my GP"* [P322, Male, age 72]	*"Have had this advice before from professional; took advice; keeps my pain under control"* [P502, Male, age 79]	*"This was pretty much the same as what I've been told so I would feel normal"* [P1517, Male, age 32]<br>*"I already received this advice. So it would make me feel reassured that I am doing the right things"* [P468, Male, age 55]
Remain active	*"I would take it on, in my experience exercising and being active has always been a good remedy for most health issues I've had in the past."* [P2183, Male, age 33]	*"Determined to be more active."* [P1065, Female, age 20]<br>*"Make me want to be healthy and exercise to keep my joints strong"* [P827, Female, age 37]	*"Yes be active"* [P369, Male, age 44]
Understanding of the cause of pain	*"good news I now know it's only muscles and how to treat it thanks"* [P229, Female, age 71]<br>*"Very good advice and gave me a detailed understanding of the cause of my pain."* [P195, Male, age 30]	*"Would make me feel comforted in knowing why there is pain in this area. As well as solutions to help alleviate it"* [P961, Female age 24]<br>*"I feel more knowledgeable with my lower back pain, what causes it and how to deal with it. I will try this advice as per the health professional"* [P289, Female, age 45]	*"Much better considering I have been told that my back problems are all related to my weight"* [P1008, Female, age 25]<br>*"I'll relax and basically know why I have lower back pain."* [P1437, Male, age 31]<br>*"It will give me great perspective what actions can I take to reduce back pain such as posture and some exercises"* [P440, Female age 58]
Challenged preconceptions	*"It would make me feel reassured but would feel like I may not be able to rest as much as I thought was necessary."* [P1599, Female, age 25]<br>*"Weird, I would be surprised I wouldn't rest"* [P1079, Female, age 23]	*"Slightly shocked, but it makes sense psychologically."* [P153, Female, age 18]	N/A

Supplementary File 5. Treatments reported for each form of advice for LBP ranked from most to least common in each group.

Guideline-based advice (n=760)	Guideline-based advice + pain science-based advice (n=727)	Guideline-based advice + ergonomic-based advice (n=733)
No treatment/stay active (n=221, 29%)	No treatment/stay active (n=208, 29%)	No treatment/stay active (n=200, 27%)
Exercise (n=132, 17%	Exercise (n=125, 17%)	Exercise (n=130, 18%)
Medication (n=95%, 13%)	Rest (n=97, 13%)	Medication (n=89, 12%)
Rest (n=69, 9%)	Medication (n=83, 11%)	Rest (n=60, 8%)
Unsure (n=58, 8%)	Unsure (n=55, 8%)	Unsure (n=48, 7%)
Manual therapy (n=37, 5%)	Physiotherapy (n=31, 4%)	Physiotherapy (n=48, 7%)
Physiotherapy (n=37, 5%)	Manual therapy (n=29, 4%)	Manual therapy (n=40, 5%)
Imaging (n=29, 4%)	Imaging (n=28, 4%)	Posture correction (n=39, 5%)
Chiropractor (n=22, 3%)	Chiropractor (n=19, 3%)	Imaging (n=31, 4%)
Lifestyle changes (n=14, 2%)	Heat (n=18, 2%)	Lifestyle changes (n=27, 4%)
Emergency department/hospital (n= 13, 2%)	Lifestyle changes (n=12, 2%)	Chiropractor (n=15, 2%)
Surgery (n=12, 2%)	Education/advice (n=12, 2%)	Heat (n=13, 2%)
Heat (n= 11, 1%)	Other treatments (n=8, 1%)	Education/advice (n=10, 1%)
Education/advice (n=11, 1%)	Emergency department/hospital (n=7, 0.96%)	Emergency department/hospital (n=8, 1%)
Specialist (n=8, 1%)	Surgery (n=7, 0.96%)	Surgery (n=8, 1%)
Cold (n=7, 0.92%)	Cold (n=6, 0.83%)	Activity modification (n=8, 1%)
Other treatments (n=6, 0.79%)	Specialist (n=5, 0.69%)	Self-management (n=6, 0.82%)
Wait and see (n=6. 0.79%)	Activity modification (n=5, 0.69%)	Acupuncture (n=6, 0.82%)
Self-management (n=5, 0.66%)	Natural/unknown therapies (n=4, 0.55%)	Other treatments (n=5, 0.68%)
Psychological therapies (n=4, 0.53%)	Wait and see (n=3, 0.41%)	Cold (n=4, 0.55%)
Posture correction (n=3, 0.39%)	Self-management (n=3, 0.41%)	Specialist (n=4, 0.55%)
Activity modification (n=3, 0.39%)	Psychological therapies (n=3, 0.41%)	Natural/unknown therapies (n=3, 0.41%)
Natural/unknown therapies (n=3, .39%)	Posture correction (n=3, 0.41%)	Psychological therapies (n=3, 0.41%)

Acupuncture (n=2, 0.26%)	Acupuncture (n=3, 0.41%)	Time off work (n=2, 0.27%)
Electrotherapy (n=1, 0.13%)	Time off work (n=2, 0.28%)	Electrotherapy (n=2, 0.27%)
Good mattress (n=1, 0.13%)	Traction (n=2, 0.28%)	Wait and see (n=1, 0.14%)
Injection (n=1, 0.13%)	Good mattress (n=1, 0.14%)	Good mattress (n=1, 0.14%)
Brace (n=1, 0.13%)	Electrotherapy (n=0, 0.00%)	Injection (n=1, 0.14%)
Immobilisation (n=1, 0.13%)	Injection (n=0, 0.00%)	Traction (n=0, 0.00%)
Time off work (n=0, 0.00%)	Brace (n=0, 0.00%)	Brace (n=0, 0.00%)
Traction (n=0, 0.00%)	Immobilisation (n=0, 0.00%)	Immobilisation (n=0, 0.00%)

n, number of participants

<1%	1% - 10%	11% - 20%	21% - 30%

Supplementary File 6. Summary of overall treatments reported for all three forms of advice.

Treatment	n=2,220
No treatment/stay active	629 (28%)
Exercise	387 (17%)
Medication	267 (12%)
Rest	226 (10%)
Unsure	161 (7%)
Physiotherapy	116 (5%)
Manual therapy	106 (5%)
Imaging	88 (4%)
Chiropractor	56 (3%)
Lifestyle changes	53 (2%)
Posture correction	45 (2%)
Heat	42 (2%)
Education/advice	33 (1%)
Emergency department/hospital	28 (1%)
Surgery	27 (1%)
Other treatments	19 (<1%)
Cold	17 (<1%)
Specialist	17 (<1%)
Activity modification	16 (<1%)
Self-management	14 (<1%)
Acupuncture	11 (<1%)
Natural or unknown therapies	10 (<1%)
Psychological therapy	10 (<1%)
Wait and see	10 (<1%)
Time off work	4 (<1%)
Electrotherapy	3 (<1%)
Good mattress	3 (<1%)
Injection	2 (<1%)
Traction	2 (<1%)
Brace	1 (<1%)
Immobilisation	1 (<1%)
