# Supplementary material for: Perceptions of advice for acute low back pain: a content analysis of qualitative data collected in a randomised experiment
Source: BMJ Open. 2024 Jul 23;14(7):e079070. doi: 10.1136/bmjopen-2023-079070 (PMC11268038; doi:10.1136/bmjopen-2023-079070)
Supplement: Supplementary file 2 [file bmjopen-2023-079070.reviewer_comments.pdf]

PEER REVIEW HISTORY

BMJ Open publishes all reviews undertaken for accepted manuscripts. Reviewers are asked to complete a checklist review form (**http://bmjopen.bmj.com/site/about/resources/checklist.pdf**) and are provided with free text boxes to elaborate on their assessment. These free text comments are reproduced below.

ARTICLE DETAILS

**TITLE (PROVISIONAL)**	Perceptions of advice for acute low back pain: A content analysis of qualitative data collected in a randomised experiment
**AUTHORS**	Augustine, Lidiya; Zadro, Joshua; Maher, Christopher; Traeger, Adrian; Jones, Caitlin; West, Courtney A; Yang, Jingjing; O'Keeffe, Mary; Jenkins, Hazel; McAuley, James; Ferreira, Giovanni

VERSION 1 – REVIEW

**REVIEWER**	Lindner, Nicole<br>University of Marburg
**REVIEW RETURNED**	26-Jan-2024

**GENERAL COMMENTS**	Thank you for the opportunity to review the manuscript “Perceptions of advice for acute low back pain: A content analysis of qualitative data collected in a randomised experiment”.<br>The topic how different forms of advices are perceived by people with acute low back pain is interesting. However, the study has some limitations.<br>One major limitation of the study is that it might be possible that the study sample is not suitable to answer questions on medical relevant back pain. Participants could be included, when they only had back pain of 1 (VAS 0-10). It is highly questionable how relevant it is to study such a minor pain at all. Many participants only had a first episode of back pain (21.2% first episode). This underpins the question how relevant it is to investigate treatment and advise at all in this sample. Maybe there is a selection bias because of the online recruitment and online survey. It might be possible that the experience of patients with more serious back pain might be different. The authors state that the experience of participants was different, when participants had the feeling the advise did not match their pain. It might be possible that the results were different in a study sample which is more typical for back pain/ has more relevant back pain.<br>Moreover, the online presentation of advise might be perceived totally different than advise by a physician in its surgery. It is unclear if the results are transferable to daily care of patients with back pain.<br>Furthermore, since nearly no difference at all was found, the results may not be of great interest to all readers.<br><br>Some points in more detail<br>Introduction<br>Explain in more detail why these three types were studied. Information on the nature of low back pain is recommended in the NICE guideline. Are ergonomic advices recommended for all patients or only for specific patients?

	Method<br>The questionnaires and questions seem to be relatively superficial. What was the reason not to use validated questionnaires e.g. HADS instead of asking a single question?<br>Did you use any method to stratify between the groups?<br>"Participants then watched a video containing the key messages for each type of advice."<br>What was the content of those videos? Can you explain it in much more detail, e.g, by using the Tidier-Checklist. Would it maybe even be possible to watch the videos? It is difficult to understand what was actually part of the three types of intervention.<br>Did you think about data saturation?<br>Did you use a software for analysis, except excel?<br>Did the two researchers analysing the data work with the rest of the team during analysis? Was there any reflecting with other researchers?<br><br>Results<br>"We recruited 2,313 participants, and 2,220 (96%) were included in the analysis"<br>Why had the rest to be excluded?<br>The mean pain intensity was 4.6. Can you provide more data? How many participants only had back pain of 1 or 2? Total range? SD?<br>Table 1, what is meant with "compensable episode"?<br>Do you present the results of the PICK-UP risk assessment tool at any point?<br>Participants also seem to mention things which do not directly link to the study "Especially when Dr says scan results are no further action required". Did you explore this further? Was there any indication that the impression of the videos was overlaid by the participants' previous experiences?<br>Did you investigate if the pain level has an influence on results? You mention that "Feelings of uncertainty and unhappiness or frustration were often expressed when participants perceived the advice did not match their pain experience". Did you also set the data gained with the questionnaires on pain intensity in relationship to the qualitative results?<br><br>Do you have any idea why many patients understood that rest could be a therapy option?<br>You state that maintaining a healthy weight was part of ergonomic advise. Often advise with regard to weight is perceived negative and might lead to a negative relationship. However, in your sample this seems not be the case. Can you reflect on that?<br>I think it is interesting that so many participants were feeling positive about the advice. Often speaking about physical activity and going to work with patients who experience pain can lead to negative emotions.

**REVIEWER**	Adje, Mishael<br>LUNEX University
**REVIEW RETURNED**	27-Mar-2024

**GENERAL COMMENTS**	I appreciate the opportunity to review this manuscript, submitted as an original article. My role as a reviewer is to assess the study's quality and relevance. Overall, I feel positive about the study. However, I do have some reservations for which I have provided suggestions.

	Introduction<br>P 4, Line 8, 9: Revise the first references (esp. cost and spending literature) reflecting the most recent and global perspective. Suggestions; Include reference: Global, regional, and national burden of low back pain, 1990–2020, its attributable risk factors, and projections to 2050: a systematic analysis of the Global Burden of Disease Study 2021. https://pubmed.ncbi.nlm.nih.gov/37273833/.<br><br>P 4, Lines 15: Make it clear from literature why clinicians would modify the guideline- recommended content and why the Authors are interested in this. The rationale needs to be clearer.<br><br>P 4, Line 39: Provide details on the similarities and discrepancies between advice on ergonomics and guideline recommendations and its potential on patient outcome.<br><br>P 4, Line 47: 'The aim of this study was to explore participants' feelings, thoughts, and expectations and their perceived need for treatment evoked by three types of advice for acute LBP'.<br>The Authors here give the impression of a primary study with a primary objective.<br><br>Method<br>P4, Line 57: Provide an original reference on the methodology<br>P5, Line 16: Exclusion criteria could be more exhaustive e.g Pregnancy, radiculopathy?<br>P5, Line 20: Could you explain how data integrity was ensured using Qualtrics since the data distribution seemed to be targeted (Table 1), was this by design? Perhaps more details on sampling and recruitment need to be provided for clarification.<br>P5, Line 31: Provide details on development or adaptation of tool for data collection.<br>P5, Line 54: How did authors confirm completion and comprehension of the video viewing tasks from all participants?<br>P5: Include the criteria used for reporting this study.<br>P6 Line 43: Discuss areas of discrepancy between coders that were resolved during analysis.<br>P6, Line 48: Could the authors explain why 'I prefer not to answer', 'having no feelings' were considered as 'irrelevant response' although assessing perception.<br>Compare P19, Line 49 and P22, Line 11- 'Irrelevant response' was coded when earlier stated it wasn't.<br><br>Results<br>P 7, Line 44: Explain the width of the most common of themes for each of the intervention category and support them using two quotes for each question.<br><br>Correct typos: P 9, line 44, also P 15, line 54 and punctuation throughout the document.<br><br>Discussion<br>P8: It would be valuable to discuss any contextual comparisons relating to country of origin or findings in this regard.<br><br><br>Supplementary files

	Resolve the following codes by providing clearer explanations or preferably merging themes. ‘Mechanism of Injury’ and ‘Aging’ and ‘Tissue damage’; ‘Hopeful’ and ‘Reassured’.<br><br>P 21, Line 33: Provide a column for explanations in Question 2 as is obtainable in Question 1<br><br>P21, Line 33: Resolve the following codes; ‘Good mattress’ and ‘Ergonomics/Posture’; ‘Immobilisation’ and ‘Taping/Bracing’; ‘No treatment’ and ‘Wait and see’; ‘Rest’ and ‘Stay healthy’ (incl. good sleep)<br><br>Supplementary file 4:<br>P 28, Line 18: Replace the theme ‘Pain experience’ or better explain it since it overlaps other themes provided.<br>P 29, Line 20: Replace the theme ‘’Avoid Treatment /Investigation’ or describe it as ‘Medication/Investigation’ to exclude physiotherapy, education.<br><br><br>We are looking forward to seeing it published.

VERSION 1 – AUTHOR RESPONSE

REVIEWER 1

1. Dear editor,
   Thank you for the opportunity to review the manuscript “Perceptions of advice for acute low back pain: A content analysis of qualitative data collected in a randomised experiment”. The topic how different forms of advices are perceived by people with acute low back pain is interesting. However, the study has some limitations.
   One major limitation of the study is that it might be possible that the study sample is not suitable to answer questions on medical relevant back pain. Participants could be included, when they only had back pain of 1 (VAS 0-10). It is highly questionable how relevant it is to study such a minor pain at all. Many participants only had a first episode of back pain (21.2% first episode). This underpins the question how relevant it is to investigate treatment and advise at all in this sample. Maybe there is a selection bias because of the online recruitment and online survey. It might be possible that the experience of patients with more serious back pain might be different. The authors state that the experience of participants was different, when participants had the feeling the advise did not match their pain. It might be possible that the results were different in a study sample which is more typical for back pain/ has more relevant back pain.

RESPONSE: The mean (SD) pain intensity of the sample was 4.6 (2) which is not dissimilar to patients recruited into trials evaluating low back pain treatment. For example in the well known STartBack trial [1] the mean pain intensities in the low and medium risk strata were ~3.5 and ~5.4.

[1] Hill JC, Whitehurst DG, Lewis M, Bryan S, Dunn KM, Foster NE, Konstantinou K, Main CJ, Mason E, Somerville S, Sowden G, Vohora K, Hay EM. Comparison of stratified primary care management for low back pain with current best practice (STarT Back): a randomised controlled trial. Lancet. 2011 Oct 29;378(9802):1560-71.

2. Moreover, the online presentation of advise might be perceived totally different than advise by a physician in its surgery. It is unclear if the results are transferable to daily care of patients with back pain.

   RESPONSE: We agree with the reviewer and have acknowledged that as a limitation in the discussion.

3. Furthermore, since nearly no difference at all was found, the results may not be of great interest to all readers.

   RESPONSE: We would respectfully disagree. We believe that the absence of differences in our study should not be factored into the decision of whether or not to publish our findings. Furthermore, not publishing the findings because there are no differences adds to the problematic issue of publication bias resulting from not publishing negative studies.

4. Introduction - Explain in more detail why these three types were studied. Information on the nature of low back pain is recommended in the NICE guideline. Are ergonomic advices recommended for all patients or only for specific patients?

   RESPONSE: Our team's systematic review [1] used meta-regression to identify whether there was a more effective type of advice (guideline, pain science, and ergonomics-based) for people with spinal pain. That review did not find a clear answer to that question. We chose to compare standard guideline messages to pain science and ergonomics messages given their popularity. Using pain science concepts is now a very popular method amongst clinicians to explain pain to patients, and messages based on ergonomic principles are often used by the general public to provide an explanation about LBP because these messages are often provided by workplaces and clinicians. We have added the following to the introduction:

   Advice based on ergonomic principles aims to educate people with acute LBP to reduce strain on the lumbar spine by maintaining good posture, practising safe lifting, and/or reducing

weight. Using pain science messages to explain pain to patients has become a very popular approach amongst clinicians; whereas, messages based on ergonomic principles are often used by the general public to make sense of LBP. Despite the popularity of these three types of advice, to date, there is very little evidence to inform clinicians on the best content to include when providing advice for acute LBP, as previous systematic reviews had shown similar outcomes for these three types of advice.(9)

[1] Jones CM, Shaheed CA, Ferreira GE, Kharel P, Christine Lin CW, Maher CG. Advice and education provide small short-term improvements in pain and disability in people with non-specific spinal pain: a systematic review. J Physiother. 2021 Oct;67(4):263-270.

5. Method - The questionnaires and questions seem to be relatively superficial. What was the reason not to use validated questionnaires e.g. HADS instead of asking a single question?

    RESPONSE: We believe the reviewer is referring to the two questions on emotional distress. We chose those two questions for two reasons:

    i. They have been previously used in low back pain research and they have been identified as contributors to healthcare seeking in people with acute low back pain [1]

    ii. Adding longer validated instruments would have added a significant number of extra questions, increasing the survey burden on participants. For example, the HADS questionnaire suggested by the reviewer has 14 questions.

    Reference

[1] Traeger AC, Hübscher M, Henschke N, Williams CM, Maher CG, Moseley GL, et al. Emotional distress drives health services overuse in patients with acute low back pain: a longitudinal observational study. European Spine Journal. 2016;25(9):2767-73.

6. Did you use any method to stratify between the groups?

    RESPONSE: We did not use any stratification method. Baseline characteristics of our sample were very balanced across the three groups as shown in Table 1.

7. "Participants then watched a video containing the key messages for each type of advice." What was the content of those videos? Can you explain it in much more detail, e.g, by using the Tidier-Checklist. Would it maybe even be possible to watch the videos? It is difficult to understand what was actually part of the three types of intervention.

    RESPONSE: Thank you for your suggestion. We have added the videos – see supplementary file 1. As the videos have been made available and they can be easily replicated we don't think the TIDieR checklist would add much value.

8. Did you think about data saturation?

RESPONSE: As this study reports data collected alongside a randomised experiment, the sample size for this study was determined by the sample size calculation based on the primary outcome reported in the primary publication. As such our sample size was fixed and therefore there were no decisions made on the basis of whether data saturation had been achieved or not. Given the large sample size of this study, we believe we collected sufficient data to draw the necessary conclusions, and collecting any further data would not have changed our findings.

9. Did you use a software for analysis, except excel?

RESPONSE: No, only Microsoft Excel.

10. Did the two researchers analysing the data work with the rest of the team during analysis? Was there any reflecting with other researchers?

RESPONSE: Members of the study team had the opportunity to comment and reflect on the results and their interpretation. We have added this to the methods.

Using the modified coding framework, all free-text responses for each question were double coded independently by two researchers (LA and GF) to confirm consistency. All coding was performed blind to the allocation of groups. The analysis was an iterative process and reflects the judgements, practices, and beliefs of a physiotherapist who has experience in qualitative research in musculoskeletal conditions and of a Master of Public Health student in qualitative research. Each response was coded using as many codes as appropriate. Members of the study team had the opportunity to comment and reflect on the results and their interpretation.

11. "We recruited 2,313 participants, and 2,220 (96%) were included in the analysis"
Why had the rest to be excluded?

RESPONSE: Among the 2,313 participants recruited into the randomised experiment, 93 did not answer the open-ended questions that are reported in this study. We have clarified in the methods and results that those participants were excluded.

Methods

For example, the code "trust in expertise" was used to capture any references to the advice/clinician being trustworthy. Participants who did not provide an answer to an open-ended question were excluded from the analysis of that question. Unusable and uninformative responses were coded as "irrelevant responses" (e.g. "I prefer not to answer").

Results

We recruited 2,313 participants, of whom 93 did not answer the open-ended questions, thus 2,220 (96%) were included in the analysis, totalling 4,440 free-text responses.

12. The mean pain intensity was 4.6. Can you provide more data? How many participants only had back pain of 1 or 2? Total range? SD?

RESPONSE: Below is the breakdown of low back pain intensity. The table and histogram below show that only 4% of participants had pain =1 and 10.3% had pain =2.

LBP intensity	Frequency	%
1	91	4.10
2	228	10.27
3	400	18.02
4	435	19.59
5	344	15.50
6	287	12.93
7	202	9.10
8	147	6.62
9	56	2.52
10	30	1.35

Total	2,220	100.00

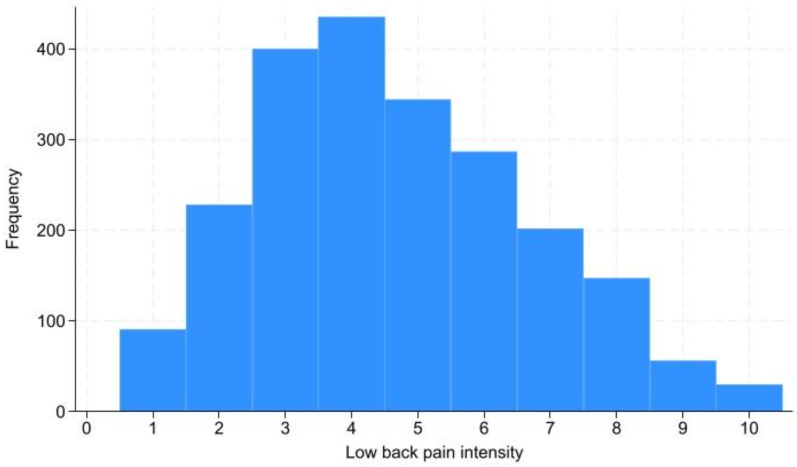

13. Table 1, what is meant with “compensable episode”?

    RESPONSE: Compensable episode is an episode that is eligible to receive compensation due to an injury sustained at work (e.g. through workers compensation). We have clarified that in the footnote in table 1.

14. Do you present the results of the PICK-UP risk assessment tool at any point?

    RESPONSE: We investigated whether the PICK-UP tool moderated the effect of the intervention on reassurance outcomes in the primary publication, and found that it didn’t. Hence we decided not to explore the PICK-UP data any further.

15. Participants also seem to mention things which do not directly link to the study “Especially when Dr says scan results are no further action required”.

    RESPONSE: For context, this is the full quote that the reviewer is referring to:

    “A bit confused! It’s hard to carry on with most normal activities when your having a lot of pain. Especially when Dr says scan results are no further action required! Yet I’m still getting bad pain! I can’t keep taking panadeine forte” [P2046, Female, age 75]

    We have re-checked the other example quotes to ensure they directly link to the study.

16. Did you explore this further? Was there any indication that the impression of the videos was overlaid by the participants' previous experiences?

    RESPONSE: Data reported in this study is from open-ended questions collected at the end of the experiment. We did not assess participants' previous experiences in depth. We have added this as a limitation in the discussion.

    We did not explore participants' previous experiences in depth, so we do not know whether the intervention changed participants' pre-existing thoughts and feelings about their LBP.

17. Did you investigate if the pain level has an influence on results? You mention that "Feelings of uncertainty and unhappiness or frustration were often expressed when participants perceived the advice did not match their pain experience". Did you also set the data gained with the questionnaires on pain intensity in relationship to the qualitative results?

    RESPONSE: No, we did not as these were not pre-planned analyses.

18. Do you have any idea why many patients understood that rest could be a therapy option?

    RESPONSE: 8%-13% of participants thought they needed rest for their LBP. These figures are similar to those reported by previous studies. For example, Hall (BMC Public Health 2021 21:682) showed that ~6% of people with back pain used bed rest as a management strategy. We have added more information on this to the discussion.

    The proportion of participants in our study who thought rest was an appropriate treatment for their LBP is similar to that reported by previous studies. For example, Hall et al. found that 6% of participants with LBP reported resting in bed to manage their pain.

19. You state that maintaining a healthy weight was part of ergonomic advise. Often advise with regard to weight is perceived negative and might lead to a negative relationship. However, in your sample this seems not be the case. Can you reflect on that?

    RESPONSE: We did not observe any negative or adverse reaction to advice to lose weight amongst participants in our study – as reflected by an absence of this theme from the free-text responses.

20. I think it is interesting that so many participants were feeling positive about the advice. Often speaking about physical activity and going to work with patients who experience pain can lead to negative emotions.

    RESPONSE: Thank you for your positive remark. It is indeed interesting and may reflect that some people can be positive and open about the idea of keeping active, although there may

still be barriers to keeping active/becoming physically active (including pain, other comorbidities, etc.).

REVIEWER 2

21. I appreciate the opportunity to review this manuscript, submitted as an original article. My role as a reviewer is to assess the study's quality and relevance. Overall, I feel positive about the study. However, I do have some reservations for which I have provided suggestions.

    RESPONSE: Thank you for your positive assessment of our paper.

22. Introduction - P 4, Line 8, 9: Revise the first references (esp. cost and spending literature) reflecting the most recent and global perspective. Suggestions; Include reference: Global, regional, and national burden of low back pain, 1990–2020, its attributable risk factors, and projections to 2050: a systematic analysis of the Global Burden of Disease Study 2021. https://pubmed.ncbi.nlm.nih.gov/37273833/.

    RESPONSE: We have included the reference suggested by the reviewer.

23. Introduction - P 4, Lines 15: Make it clear from literature why clinicians would modify the guideline- recommended content and why the Authors are interested in this. The rationale needs to be clearer.

    RESPONSE: Thank you for your suggestion. Reviewer 1 made a similar comment – please refer to comment #6 for more information.

24. Introduction - P 4, Line 39: Provide details on the similarities and discrepancies between advice on ergonomics and guideline recommendations and its potential on patient outcome.

    RESPONSE: We have added the following information to the introduction:

    The key contrast between advice based on ergonomic principles and guideline advice is the biomechanical framework used (in ergonomic advice) to explain how back pain develops and may persist.

25. P 4, Line 47: 'The aim of this study was to explore participants' feelings, thoughts, and expectations and their perceived need for treatment evoked by three types of advice for acute LBP'. The Authors here give the impression of a primary study with a primary objective.

RESPONSE: We have revised the aims:

The aim of this study was to explore participants' feelings, thoughts, and expectations and their perceived need for treatment evoked by three types of advice for acute LBP using qualitative data collected alongside a randomised experiment.

26. Method - P4, Line 57: Provide an original reference on the methodology

    RESPONSE: We have added the reference to the published randomised experiment (see comment #2 for more details on the reference).

27. P5, Line 16: Exclusion criteria could be more exhaustive e.g Pregnancy, radiculopathy?

    RESPONSE: The study describes the inclusion and exclusion criteria used to determine eligibility for the study. We cannot add criteria that weren't used in the study.

28. P5, Line 20: Could you explain how data integrity was ensured using Qualtrics since the data distribution seemed to be targeted (Table 1), was this by design? Perhaps more details on sampling and recruitment need to be provided for clarification.

    RESPONSE: We added more information about Qualtrics in the methods.

    Participation is incentivised using points which participants can redeem for cash, airline miles, and gift cards. Qualtrics implements numerous strategies to avoid duplication (eg, IP address checks) and ensure validity of responses (eg, Captcha verification to screen out bots, flagging speeders or suspicious open-ended responses).(12, 13)

29. P5, Line 31: Provide details on development or adaptation of tool for data collection.

    RESPONSE: We presume that the reviewer is referring to the survey used to collect the data for this study. The survey was adapted from previous studies published by our group, including the open-ended questions that inform this manuscript. We have added additional information in the methods.

References:

- Zadro JR, Ferreira GE, Muller R, Sousa Filho LF, Malliaras P, West CA, et al. Education can reassure people with rotator cuff-related shoulder pain: a 3-arm, randomised, online experiment. Pain. 2024;165(4):951-8.
- Zadro JR, O'Keeffe M, Ferreira GE, Haas R, Harris IA, Buchbinder R, et al. Diagnostic Labels for Rotator Cuff Disease Can Increase People's Perceived Need for Shoulder Surgery: An Online Randomized Controlled Trial. J Orthop Sports Phys Ther. 2021;51(8):401-11.
- Zadro JR, O'Keeffe M, Ferreira GE, Traeger AC, Gamble AR, Page R, et al. Diagnostic labels and advice for rotator cuff disease influence perceived need for shoulder surgery: an online randomised experiment. J Physiother. 2022;68(4):269-76.
- O'Keeffe M, Michaleff ZA, Harris IA, Buchbinder R, Ferreira GE, Zadro JR, et al. Public and patient perceptions of diagnostic labels for non-specific low back pain: a content analysis. Eur Spine J. 2022.
- Joshua RZ, Zoe AM, Mary O, Keeffe, Giovanni EF, Romi H, et al. How do people perceive different labels for rotator cuff disease? A content analysis of data collected in a randomised controlled experiment. BMJ Open. 2021;11(12):e052092.

30. P5, Line 54: How did authors confirm completion and comprehension of the video viewing tasks from all participants?

RESPONSE: We assessed how long participants stayed on the video in the survey but did not assess comprehension. We have added this information to the manuscript.

RESULTS

We recruited 2,313 participants, of whom 93 did not answer the open-ended questions, thus 2,220 (96%) were included in the analysis, totalling 4,440 free-text responses. The participants in the three groups had similar characteristics (Table 1). Participants were on average 38.4 years old and 58.2% were female. 21.2% (n= 492) of participants reported no previous history of LBP; 19.1% (n= 444) of participants reported previous history of LBP and had received advice for LBP. The mean (SD) LBP intensity was 4.6 (2). We coded 317 (14.3%) responses as irrelevant (e.g. "prefer not to answer"). Participants spent a median (interquartile range) of 10 minutes (7.2 to 14.6) on the survey and watched the videos for an average of 88% (19.3) of their total duration.

31. P5: Include the criteria used for reporting this study.

RESPONSE: Apologies, but we could not understand exactly what the reviewer is referring to. If the reviewer is referring to a reporting checklist, there isn't a specific checklist developed that focuses on our type of study. Available checklists for qualitative research (e.g. COREQ) are not applicable; for example, they contain multiple questions focusing on details of interviews and relationship established between researchers and participants – concepts that are not applicable to our manuscript.

32. P6 Line 43: Discuss areas of discrepancy between coders that were resolved during analysis.

    RESPONSE: Discrepancy between coders were resolved in meetings where both coders explained their decision and consensus was reached. We did not collect data that allows us to answer this question.

33. P6, Line 48: Could the authors explain why 'I prefer not to answer', 'having no feelings' were considered as 'irrelevant response' although assessing perception.

    RESPONSE: We judged that those two examples of responses would not have addressed the questions being asked in a meaningful way and were therefore considered to be irrelevant:

- "If your health professional gave you this advice, how would it make you feel?"
- "If your health professional gave you this advice, what treatments (if any) do you think you would need?"

34. Compare P19, Line 49 and P22, Line 11- 'Irrelevant response' was coded when earlier stated it wasn't.

    RESPONSE: On page 7 of 32 (L49) it was stated that: "Unusable and uninformative responses were coded as "irrelevant responses" (e.g. "I prefer not to answer").", and on page 8 of 32 it was stated that: "We coded 317 (14.3%) responses as irrelevant (e.g. "prefer not to answer")." In both instances where "irrelevant response" was mentioned in text we did state that they were coded.

35. Results - P 7, Line 44: Explain the width of the most common of themes for each of the intervention category and support them using two quotes for each question.

    RESPONSE: The data requested by the reviewer are available in the supplementary files. The tables contain comprehensive data showing the number and proportion of codes stratified by type of advice. Supplementary file 5 contains a large number of quotes to support interpretation of our findings. Adding more quotes to the manuscript would substantially increase the manuscript's word count which we are not in a position to do.

36. Correct typos: P 9, line 44, also P 15, line 54 and punctuation throughout the document.

RESPONSE: Below is the sentence on P9L44:

P9L44: These findings are similar to those from a recent randomised experiment which compared guideline-based advice with a structure-based treatment recommendation for people with shoulder pain. That study found participants receiving guideline-based advice more often expressed feelings of reassurance and having a minor issue compared to participants receiving a structure-based treatment recommendation.

P15 on our manuscript only contains references. We are not clear what punctuation needs to be corrected throughout the manuscript. We are happy to work with the editors and journal's production team to address these issues.

37. Discussion - P8: It would be valuable to discuss any contextual comparisons relating to country of origin or findings in this regard.

RESPONSE: Stratifying data by country of origin (assuming that the reviewer is talking about country of birth) would have been impossible because we did not collect these data. We only collected data on where participants were living at the time of completing the survey.

38. Supplementary files - Resolve the following codes by providing clearer explanations or preferably merging themes. 'Mechanism of Injury' and 'Aging' and 'Tissue damage'; 'Hopeful' and 'Reassured'.

RESPONSE: We do not understand what the reviewer means by asking us to 'resolve codes'. The coding framework was developed using a standardised process and was agreed upon by the study investigators. We do not feel like it can be changed at this stage.

39. P 21, Line 33: Provide a column for explanations in Question 2 as is obtainable in Question 1

RESPONSE: We believe the table where descriptions of treatments for question 2 are provided in the supplementary file is straightforward and does not need an additional column as requested by the reviewer. That table lists the codes and provides examples of treatments that were considered under those codes.

40. P21, Line 33: Resolve the following codes; ‘Good mattress’ and ‘Ergonomics/Posture’; ‘Immobilisation’ and ‘Taping/Bracing’; ‘No treatment’ and ‘Wait and see’; ‘Rest’ and ‘Stay healthy’ (incl. good sleep)

RESPONSE: Please see response to comment #40.

41. Supplementary file 4: P 28, Line 18: Replace the theme ‘Pain experience’ or better explain it since it overlaps other themes provided.

RESPONSE: Please see response to comment #40.

42. P 29, Line 20: Replace the theme ‘’Avoid Treatment /Investigation’ or describe it as ‘Medication/Investigation’ to exclude physiotherapy, education.

RESPONSE: Please see response to comment #40.

VERSION 2 – REVIEW

**REVIEWER**	Adje, Mishael<br>LUNEX University
**REVIEW RETURNED**	07-Jun-2024

**GENERAL COMMENTS**	Thanks for inviting me for a second review. Thank you to the authors for making the corrections to improve the manuscript. It has become clearer, which is appreciated. However, some comments still need to be addressed to enhance the transparency and relevance of the manuscript. My comments are provided below.<br><br>-------------<br>1. Results: P 7, Line 40: The authors said 'Participants spent a median (interquartile range) of 10 minutes (7.2 to 14.6) on the survey and watched the videos for an average of 88% (19.3) of their total duration.'<br><br>It is quite important that the authors could prove the patients understood the message delivered through these videos. Their responses were based on this, and the content of the messages was quite specific and needed some attention to have relevant opinions. Time alone does not suffice. Some studies have included the critical component of comprehension with follow-up interviews, open-ended questions, tailored/interactive videos, embedded quizzes, etc (Hill et al., 2011, Adje et al., 2023). Address what extent was this considered in your study and its possible impact.

	3. P6 Line 55: The authors had responded to my question: 'Discuss areas of discrepancy between coders that were resolved during analysis - RESPONSE: Discrepancy between coders were resolved in meetings where both coders explained their decision and consensus was reached. We did not collect data that allows us to answer this question.'<br><br>Please clarify if this (no notes on the iterative process) was an omission or part of the design. It would improve the manuscript if the analytical process or result could be clearer. For example, the following below are unclear:<br><br>- The theme was labelled ‘Pain experience’ but not including psychological distress (already given in a separate theme).<br>- The theme ’Avoid Treatment /Investigation’ is not obvious to readers that authors excluded physiotherapy as well as CBT, education.<br>- The theme ‘Immobilisation’ includes sling but not including ‘Taping/Bracing’ coded separately.<br>- The theme ‘Rest’ is treated differently from ‘Stay healthy’ (incl. good sleep), reduce stress.<br>- Psychological therapy including CBT is coded separately from physiotherapy.<br>- ‘No treatment’ is coded separately from ‘wait and see - no treatment unless pain got worse’.<br><br>Please provide clearer explanations for these codes.<br><br>4. Discussion - P8: The authors had responded to my question: 'It would be valuable to discuss any contextual comparisons relating to country of origin or findings in this regard. RESPONSE: Stratifying data by country of origin (assuming that the reviewer is talking about country of birth) would have been impossible because we did not collect these data. We only collected data on where participants were living at the time of completing the survey.'<br><br>Page 5 Line 48: ‘Participants provided demographic information at the start of the questionnaire. This included data on…country of birth’. Not sure if this meant pilot study only, but clarify why this data was omitted from your sociodemographic Table 1. Nonetheless, please include contextual findings from what was obtained to enhance your discussion.

VERSION 2 – AUTHOR RESPONSE

1. Results: P 7, Line 40: The authors said 'Participants spent a median (interquartile range) of 10 minutes (7.2 to 14.6) on the survey and watched the videos for an average of 88% (19.3) of their total duration.'

It is quite important that the authors could prove the patients understood the message delivered through these videos. Their responses were based on this, and the content of the messages was quite specific and needed some attention to have relevant opinions. Time alone does not suffice. Some studies have included the critical component of comprehension with follow-up interviews, open-

ended questions, tailored/interactive videos, embedded quizzes, etc (Hill et al., 2011, Adje et al., 2023). Address what extent was this considered in your study and its possible impact.

RESPONSE: Thank you for this comment. Unfortunately data for our study was collected only once, and there were no follow-up interviews that could address the reviewer's suggestions. This is a limitation of the study, which we have now highlighted in the discussion.

Our study has limitations. Our experiment was conducted online, and findings may differ had the experiment been conducted in patients seeking care in the real world. We collected outcome data at one point in time, immediately after participants were given the advice. Our findings may have been different as participants reflected further over time. Advice in our study was delivered by a physiotherapist via brief pre-recorded videos. Our findings may have been different if the advice had been delivered by a health professional (e.g. general practitioner, physiotherapist) during a clinical encounter. We did not include non-English speaking participants, which limits the generalisability of findings to linguistically diverse populations. Our sample was overall well educated and had high health literacy. We did not explore participants' previous experiences in depth, so we do not know whether the intervention changed participants' pre-existing thoughts and feelings about their LBP. We also did not explore whether participants understood the messages that were delivered to them in each group.

3. P6 Line 55: The authors had responded to my question: 'Discuss areas of discrepancy between coders that were resolved during analysis - RESPONSE: Discrepancy between coders were resolved in meetings where both coders explained their decision and consensus was reached. We did not collect data that allows us to answer this question.'

Please clarify if this (no notes on the iterative process) was an omission or part of the design. It would improve the manuscript if the analytical process or result could be clearer. For example, the following below are unclear:

- The theme was labelled 'Pain experience' but not including psychological distress (already given in a separate theme).
- The theme 'Avoid Treatment /Investigation' is not obvious to readers that authors excluded physiotherapy as well as CBT, education.
- The theme 'Immobilisation' includes sling but not including 'Taping/Bracing' coded separately.
- The theme 'Rest' is treated differently from 'Stay healthy' (incl. good sleep), reduce stress.
- Psychological therapy including CBT is coded separately from physiotherapy.
- 'No treatment' is coded separately from 'wait and see - no treatment unless pain got worse'.

Please provide clearer explanations for these codes.

RESPONSE: Notes were taken by the coders and these were used to inform the discussion to agree on codes. We have clarified that in the methods.

Where there was disagreement, notes were compared and disagreements between coders were discussed and resolved by consensus. We used Microsoft Excel v16.54 to conduct our statistical analysis. Frequencies of codes were used to determine the prominence of themes. The coding framework for each question is provided in supplementary file 2.

We fail to understand what the reviewer wants us to do by suggesting we provide 'clearer explanations for the codes'. The codes are self-explanatory. We recognise that there may be disagreements about how our data were coded. However the proposed suggestions do not seem reasonable as the reviewer seems to be suggesting that we combine different codes that are not meant to be combined. We would prefer not to act on this request for a number of reasons.

First, because, as described in the previous round of peer-review, the coding process was undertaken following well described methods, it involved hours of work on our end, discussions, and consensus meetings. It is not customary that peer-reviewers suggest codes and themes to be modified, even more so when they do not have in-depth knowledge of the data.

The second problem that we see is that the reviewer's suggestions are problematic. For example, psychological therapy including CBT is clearly different to physiotherapy; use of sling for immobilisation is clearly different to using taping or bracing which allow patients to still move (i.e. they are not immobilised). No treatment is different than wait-and-see, and so on.

4. Discussion - P8: The authors had responded to my question: 'It would be valuable to discuss any contextual comparisons relating to country of origin or findings in this regard. RESPONSE: Stratifying data by country of origin (assuming that the reviewer is talking about country of birth) would have been impossible because we did not collect these data. We only collected data on where participants were living at the time of completing the survey.'

Page 5 Line 48: 'Participants provided demographic information at the start of the questionnaire. This included data on…country of birth'. Not sure if this meant pilot study only, but clarify why this data was omitted from your sociodemographic Table 1. Nonetheless, please include contextual findings from what was obtained to enhance your discussion.

RESPONSE: The reviewer is correct, we did collect data on country of birth as reported in the methods. Presenting information stratified by country of birth is however not going to be informative as there are over 68 reported countries of birth. We do agree that investigating cultural differences in how advice is perceived is an interesting research question, but that is not a question that our study can answer.

1. Afghanistan
2. Albania
3. Argentina
4. Australia
5. Bangladesh

6. Bhutan
7. Brazil
8. Canada
9. Chile
10. China
11. Colombia
12. Croatia
13. Cuba
14. Cyprus
15. Denmark
16. Dominican Republic
17. Ecuador
18. Egypt
19. El Salvador
20. Fiji
21. France
22. Gabon
23. Germany
24. Guyana
25. Haiti
26. Hong Kong
27. Hungary
28. India
29. Indonesia
30. Iran
31. Ireland
32. Italy
33. Jamaica
34. Japan
35. Kenya
36. Kuwait
37. Lebanon
38. Malaysia
39. Mauritius
40. Mexico
41. Morocco
42. Nepal
43. Netherlands
44. New Zealand
45. Nigeria
46. Pakistan
47. Philippines
48. Poland
49. Portugal
50. Romania
51. Russia
52. Samoa
53. Singapore
54. Sprain
55. South Korea
56. Sri Lanka
57. Suriname
58. Switzerland
59. Taiwan
60. Thailand
61. Turkey
62. UAE
63. Ukraine
64. United Kingdom
65. United States

66. Venezuela
67. Vietnam
68. Zambia

Reviewer: 2
Competing interests of Reviewer: None

VERSION 3 – REVIEW

**REVIEWER**	Adje, Mishael<br>LUNEX University
**REVIEW RETURNED**	24-Jun-2024

**GENERAL COMMENTS**	None
